# Curvature‐Assisted Vesicle Explosion Under Light‐Induced Asymmetric Oxidation

**DOI:** 10.1002/advs.202400504

**Published:** 2024-08-13

**Authors:** Vinit Kumar Malik, On Shun Pak, Jie Feng

**Affiliations:** ^1^ Department of Mechanical Science and Engineering University of Illinois Urbana‐Champaign Urbana IL 61801 USA; ^2^ Department of Mechanical Engineering and Department of Applied Mathematics Santa Clara University Santa Clara CA 95053 USA; ^3^ Materials Research Laboratory University of Illinois Urbana‐Champaign Urbana IL 61801 USA

**Keywords:** biomedical engineering, light‐induced membrane oxidation, lipid vesicles, membrane modeling, spontaneous curvature

## Abstract

Exposure of cell membranes to reactive oxygen species can cause oxidation of membrane lipids. Oxidized lipids undergo drastic conformational changes, compromising the mechanical integrity of the membrane and causing cell death. For giant unilamellar vesicles, a classic cell mimetic system, a range of mechanical responses under oxidative assault has been observed including formation of nanopores, transient micron‐sized pores, and total sudden catastrophic collapse (i.e., explosion). However, the physical mechanism regarding how lipid oxidation causes vesicles to explode remains elusive. Here, with light‐induced asymmetric oxidation experiments, the role of spontaneous curvature on vesicle instability and its link to the conformational changes of oxidized lipid products is systematically investigated. A comprehensive membrane model is proposed for pore‐opening dynamics incorporating spontaneous curvature and membrane curling, which captures the experimental observations well. The kinetics of lipid oxidation are further characterized and how light‐induced asymmetric oxidation generates spontaneous curvature in a non‐monotonic temporal manner is rationalized. Using the framework, a phase diagram with an analytical criterion to predict transient pore formation or catastrophic vesicle collapse is provided. The work can shed light on understanding biomembrane stability under oxidative assault and strategizing release dynamics of vesicle‐based drug delivery systems.

## Graphical abstract

Membrane destabilization via light‐induced lipid oxidation has ramifications for cell survivability and remedial potential. Here, a mechanistic model is presented that links conformational changes occurring at the molecular level during oxidation to multiple destabilization dynamics of lipid vesicles. The model successfully predicts a phase diagram for vesicle explosion under oxidation, and might pave the way to understand stability of inherently asymmetric bio‐membranes subject to oxidative insults and can guide the design of vesicle‐based precision delivery systems.

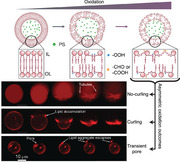

## 1 Introduction

Biological membranes are continually exposed to diverse environmental assaults which threaten membrane stability. The shape and structure of phospholipids, one of the main constituents of cell membranes,^[^ ^1^, ^2^ ^]^ play a crucial role in membrane physiology.^[^ ^3^, ^4^ ^]^ Exposure to reactive oxygen species (ROS) which serves an essential role in cell signaling and survival, concomitantly, represents an inexorable threat to catastrophically altering the physicochemical properties of membrane phospholipids.^[^ ^5^, ^6^ ^]^ Cells have evolved intricate anti‐oxidant mechanisms to mitigate elevated concentrations of ROS^[^ ^7^ ^]^ and failure of this machinery results in deleterious lipid oxidation,^[^ ^8^ ^]^ a process implicated in multiple disease pathologies.^[^ ^9^ ^]^ Additionally, phospholipid oxidation has also been leveraged as the primary mechanism of photodynamic therapy, leading to the targeted destruction of malignant cells.^[^ ^10^, ^11^ ^]^ The dramatic conformational changes induced by phospholipid oxidation may be a potential source of oxidative membrane destabilization as it alters membrane properties such as fluidity, bending rigidity, and permeability.^[^ ^12^, ^13^ ^]^ In particular, permeabilization, a puncturing in the cell membrane resulting in loss of specificity in solute transport across membrane, is a frequent mode of membrane integrity loss, leading to dire outcomes for the cell.^[^ ^10^, ^11^ ^]^ It is then perhaps unsurprising that cell membrane permeabilization, driven by lipid oxidation, has become a topic of considerable interest as a result of its significant implications for cell viability and therapeutic potential.^[^ ^14^, ^15^, ^16^ ^]^

**Figure 1:**
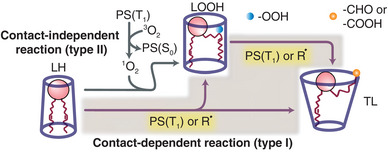
Schematic of lipid oxidation by a photosensitizer (PS), including the contact‐independent (type II) and contact‐dependent (type I, shaded region) reactions. PS(S_0_), PS(T_1_): PS in the ground, and triplet states; ^3^O_2_, ^1^O_2_: oxygen in the ground, and singlet state; R^.^: general radical species; LH: unoxidized lipid; LOOH: lipid hydroperoxides; TL: truncated lipids. Blue, and orange spheres represent polar groups –OOH and –CHO or –COOH, respectively. The outlines around the lipids show their molecular geometry. LH: inverted conical, LOOH: cylindrical, TL: conical.

Photosensitization of giant unilamellar vesicles (GUVs) has emerged as an indispensable approach in the pursuit of identifying the causal role of lipid oxidation on membrane stability.^[^ ^14^ ^]^ Photosensitization is a means to induce oxidative damage through the generation of ROS via light absorption by a photosensitizer (PS) molecule. As illustrated in **Figure** **1**, in the presence of oxygen (^3^O_2_), the photosensitization reactions can occur in two ways: contact‐independent (type II) or contact‐dependent (type I). In the contact‐independent pathway, a singlet oxygen (^1^O_2_), generated by the excited triplet state of photosensitizer (PS(T_1_)), attacks an unsaturated site of lipids to form lipid hydroperoxides (LOOH) through the addition of a polar group (–OOH). In the contact‐dependent pathway (shaded region in Figure 1), the reaction proceeds through a direct collision of PS(T_1_) or a general radical R^.^ with an unoxidized lipid (LH) or an LOOH. This triggers hydrogen abstraction of the lipid double bond, leading to additional oxidation products, including an oxidatively truncated lipid (TL) with a tail end bearing an aldehyde (─CHO) or carboxyl (─COOH) group, along with ketones, alcohols, and cleaved lipid fragments of shortchain hydrocarbons.^[^ ^17^, ^18^, ^19^ ^]^ In a lipid bilayer, the polar groups added to lipids during lipid oxidation migrate toward the membrane‐water interface, inducing substantial conformational changes. Notably, as shown in Figure 1, LOOH adopts a cylindrical shape and has a monolayer spontaneous curvature ≈0.^[^ ^20^, ^21^ ^]^ In contrast, TL adopts a conical shape and exhibits a positive monolayer spontaneous curvature >0.^[^ ^20^, ^22^, ^23^ ^]^

As a result, the reorientation of polar moieties disrupts lipid packing^[^ ^24^, ^25^ ^]^ and monolayer spontaneous curvature of oxidized lipids, generating packing stress within the larger bilayer structure.^[^ ^26^ ^]^ Relaxation of packing stress is considered to drive the redistribution of lipids in the membrane, inducing morphological changes such as budding and invaginations, and even expulsion of oxidized lipids or cleaved fragments from lipid bilayers, culminating in membrane destabilization. Distinct modes of membrane rupture in vesicles driven by lipid oxidation have been reported as the formation of nanopores,^[^ ^17^, ^19^, ^22^, ^23^, ^27^, ^28^, ^29^, ^30^ ^]^ micron‐sized pores,^[^ ^18^, ^25^, ^30^, ^31^, ^32^ ^]^ and even catastrophic membrane collapse.^[^ ^33^ ^]^

Redistribution of lipids through lateral diffusion and aggregation of conical lipids (monolayer spontaneous curvature >0) have been suggested to favor nanopore formation.^[^ ^28^, ^29^ ^]^ In addition, it has been suggested that the loss of membrane area in the form of budding events, or vesicle swelling from osmotic imbalance due to the accumulation of released oxidation products inside the vesicle, can generate membrane tension sufficient to form micron‐sized pores.^[^ ^18^, ^25^ ^]^ The focus of prior studies has been mainly on understanding the mechanism and kinetics of lipid oxidation through leakage assays of vesicles. Although considerable progress has been made in understanding the chemical pathways of lipid oxidation, the role of oxidation‐induced disruption of lipid monolayer spontaneous curvature and the resulting stress on membrane mechanical stability remains largely unexplored. Furthermore, the conditions favoring the unrecoverable sudden total collapse of a vesicle, an event we refer to as explosion, are still elusive.

With systematic experiments and modeling, we develop a mechanical description of how oxidation‐induced conformational changes determine the stability and integrity of lipid vesicles. We show how the asymmetric distribution of PS leads to unequal rates of oxidation at inner and outer leaflets, creating non‐monotonic changes in spontaneous curvature and induction of packing stress. We account for different modes of membrane rupture including vesicle collapse with and without accumulation of lipids around the pore rim. Using this framework, we are able to elucidate why vesicle explosion appears possible only in the early stages of oxidative processes and only transient pores emerge at longer times. We conclude with a phase diagram delineating vesicle explosion and pore formation, both numerically and analytically. This comprehensive framework unifies prior experimental observations and highlights the role of spontaneous curvature in maintaining membrane mechanical stability.

## 2 Results

### 2.1 Asymmetric PS Distribution across a Membrane Causes Asymmetric Oxidation of the Lipid Bilayer

**Figure 2:**
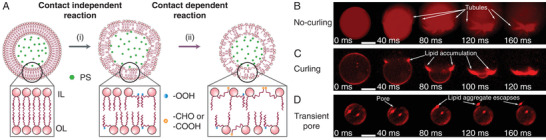
Asymmetric oxidation of a GUV encapsulating a photosensitizer (PS) and modes of membrane rupture observed in experiments. A) Morphological modifications of a GUV encapsulating PS undergoing photosensitized oxidation. The inset shows the distribution of lipid oxidation products in the lipid bilayer. Initially the vesicle membrane maintains a symmetric configuration of unoxidized lipids (LH). i) In the contact independent reaction pathway, lipids oxidize symmetrically across the membrane to produce LOOH (with a cylindrical shape) with an addition of ─OOH (blue circle). ii) In the contact dependent reaction pathway, LH and LOOH further oxidize to form truncated lipids (with a conical shape) bearing a polar group at the truncated tail (orange circle: an aldehyde (─CHO) or a carboxyl (─COOH)). Asymmetric oxidation leads to asymmetric distribution of oxidized lipid products in inner (IL) and outer (OL) leaflets. B–D) Modes of membrane rupture for GUVs (encapsulated PS concentration *c* = 20 mM), showing B) no‐curling, C) curling, and D) pore. In the no‐curling mode (B) of vesicle explosion, the sprouting of tubules‐like structures around the rim with no accumulation is observed. Meanwhile, in the curling mode (C) of vesicle explosion, a bright spot showing lipid accumulation around the pore rim grows as the explosion progresses. In other cases, a transient pore forms and reseals (D) without vesicle explosion. Here, *t* = 0 is defined as the moment when membrane rupture is observed. In (B–D), the fluorescent signal could be a mixed signal from the lipid label and HPTS. Additionally, we note that HPTS is not expected to be absorbed inside the membrane layer due to the polar nature and high solubility of HPTS in aqueous solutions.^[^ ^34^, ^35^ ^]^ Scale bars are 10 µm.

To understand how membrane oxidation impacts membrane integrity and stability, we fabricated GUVs from 1,2‐dioleoyl‐sn‐glycero‐3‐phosphocholine (DOPC), encapsulating PS (8‐Hydroxypyrene‐1,3,6‐trisulfonic acid trisodium salt (HPTS)), as shown in **Figure** **2**A (only PS molecules are shown for clarity, see Experimental Section for the vesicle fabrication). A general scheme in Figure 1 shows how the lipid oxidation advances via contact‐independent and contact‐dependent pathways. The HPTS, which is the PS in our current experiments, is highly soluble in aqueous solutions with a neutral pH^[^ ^34^ ^]^ with a logP value of <−3,^[^ ^35^ ^]^ suggesting the preferred location of PS molecules is on the surface of inner leaflet instead of inside of the lipid membrane. The PS molecules are also impermeable to the membrane.^[^ ^36^ ^]^ In previous experiments with DOPC vesicles where PS was located at the surface of inner and outer leaflets, it has been shown that the contact‐independent reactions occur an order of magnitude faster than the contact‐dependent reactions.^[^ ^18^ ^]^ Accordingly, lipid oxidation is conceived to transpire as a two‐step consecutive reaction: formation of LOOH via contact‐independent pathway followed by formation of TL and other oxidation products via a contact‐dependent pathway (LH ⟶ LOOH ⟶ TL).^[^ ^14^, ^18^, ^37^ ^]^ Therefore, we believe that such a two‐step lipid oxidation will be applicable in our experiments. Figure 2A shows a GUV response upon irradiation in the presence of a PS. At the beginning of irradiation (Figure 2A–i), following the formation of LOOH, the vesicle membrane expands as the membrane gains area and high amplitude fluctuations of the bilayer are visible. In this step, lipid oxidation is mediated by singlet oxygen attacks to form LOOH (see Figures 1 and 2A–i). We note that the lifetime of singlet oxygen molecules in lipid bilayer ≈10 µs is significantly longer than the lifetime of ≈4 µs in aqueous solutions.^[^ ^38^, ^39^ ^]^ In addition, it was shown that only ≈20% of singlet oxygen molecules react with the unsaturated lipid.^[^ ^40^ ^]^ Therefore, considering the relatively low reaction efficiency singlet oxygen with the lipids, and the ability of singlet oxygen to permeate through the membrane and to diffuse relatively large distances (≈100 nm during the lifetime of ≈4 µs in aqueous solutions), we believe that singlet oxygen molecules arriving at inner leaflet could reach and react with the unsaturated lipids in the outer leaflet. As a result, a direct contact between a PS molecule and membrane is not required for contact‐independent reactions to proceed.^[^ ^15^, ^16^, ^19^ ^]^ Consequently, LOOH distribution remains symmetrical in inner and outer leaflets (see Figure 2A–i). In our experiment, the vesicle deformations and shape fluctuations (marker of contact‐independent type II oxidation^[^ ^17^, ^18^, ^33^ ^]^) disappeared in about a second of light exposure, indicating the completion of contact‐independent reactions (see Figure S3 and Movie S1, Supporting Information).

Next, contact‐dependent reactions lead to further oxidation of LOOH to form TL (see Figures 1 and 2A–ii). In this contact‐dependent pathway, a physical contact between PS(T_1_) and its target is required. Even in the absence of PS outside of the vesicle initially, slow leakage of PS through spontaneously formed nanopores could sustain the contact‐dependent reactions at the outer leaflet.^[^ ^17^, ^19^, ^29^ ^]^ Owing to PS dissolution in the aqueous environment and PS molecules impermeability to the lipid membrane, the proximity of the PS and its affinity toward the lipid membrane is a determinant for the rate and extent of the contact‐dependent reactions.^[^ ^15^ ^]^ Consequently, oxidation occurs more rapidly at the inner leaflet because of excess PS molecules in close proximity of the inner leaflet. This discrimination in oxidation reaction rates creates an asymmetric distribution of lipid oxidation products across inner and outer leaflets (see Figure 2A–ii), which results in a spontaneous curvature $H_{s}$,^[^ ^41^ ^]^ conceptualized as the preferred curvature a lipid bilayer adopts under stress‐free conditions. The incompatibility between spontaneous curvature and the vesicle membrane curvature induces an energetic penalty impacting membrane stability. In the following sections, we will discuss how the spontaneous curvature $H_{s}$ generated from asymmetric oxidation of the lipid vesicle plays an important role in determining membrane integrity and stability.

### 2.2 Modes of Membrane Rupture

Prior experimental studies have suggested the disruption in oxidation‐induced lipid packing and generation of spontaneous curvature, driving the budding of small vesicles and tubule‐like structures from the vesicle membrane.^[^ ^17^, ^30^, ^42^ ^]^ Aligned with this view, we observe tubule‐like structures budding off the vesicles in our experiments (see Movies S2 and S3, Supporting Information). The loss in membrane area from these budding events causes membrane area strain, resulting in the build‐up of the membrane tension. Once the critical membrane tension is achieved, the membrane ruptures and opens a pore. The relaxation of membrane stretching energy $E_{s}$ drives the initial pore growth. Distinctly in our experimental system, we recognize three modes of membrane rupture: explosion with no‐curling, explosion with curling, and transient pore formation (see Figure 2B–D). In vesicles explosion with no‐curling (Figure 2B), small tubules can be seen sprouting from the retracting pore edge. Tubules subsequently merge into a single large cluster of lipid aggregate. These vesicles collapse within ≈100 ms once the pore opens. This mode of membrane rupture has been reported in the case of multicomponent charged membranes, where spontaneous curvature has also been suggested to impact vesicle destabilization.^[^ ^43^ ^]^ In contrast, vesicle explosion with curling (Figure 2C) produces a very bright spot at the pore edge, which keeps growing as the pore expands, indicating material accumulation along the edge. Eventually, the membrane flattens and transforms into a lipid aggregate cluster. In a subset of observations, the lipid membrane ruptures with a series of transient pores without a total irreversible collapse (Figure 2D). The formation of micron‐sized pores could be observed optically under a microscope due to their relatively large size. Moreover, we use the release of lipid aggregate confined inside of a vesicle to mark a pore opening. In Figure 2D, a lipid aggregate, initially confined inside of a vesicle during the fabrication, escapes through the transient pore. The pore reseals quickly before the second lipid aggregate could escape. While we expect that the formation of spontaneous nanopores is possible due to oxidized lipids diffusion and aggregation in our experiments, this mechanism could not account for the formation of micron‐sized pores or vesicle explosion. Therefore, in this work, we focus on the impact of lipid oxidation on vesicle stability regarding oxidation‐induced micron‐sized pore opening or vesicle explosion. Markedly, in all modes of rupture, only a single micron‐sized pore was observed.

### 2.3 Impact of PS Concentration Difference on Membrane Rupture

**Figure 3:**
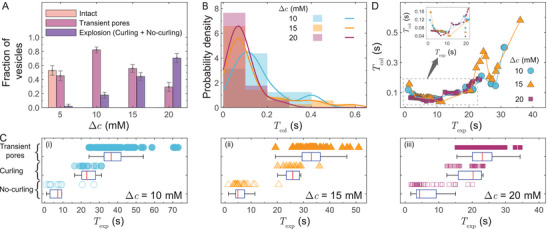
Vesicle rupture and explosion statistics. A) Fraction of vesicles responding in different modes during the light illumination. No vesicle survives at higher PS concentration differences (Δc= 10, 15, and 20 mM). Error bars represent the standard error of a proportion,^[^ ^44^ ^]^ with negative errors truncated to zero. B) Probability density of vesicle collapse time, $T_{\mathrm{col}}$, at different Δc. C) Vesicle exposure time, $T_{\exp}$, for PS concentration differences Δc= 10, 15, and 20 mM. The box‐plot represents the distribution of $T_{\exp}$ based on first quartile, median (red line), and third quartile.^[^ ^45^ ^]^ The whiskers extend to the minimum and maximum values of $T_{\exp}$ excluding outliers. For a given Δc, the median values of $T_{\exp}$ increases across the modes of no‐curling, curling, and transient pores, respectively. D) Non‐monotonic evolution of Gaussian‐weighted moving average $T_{\mathrm{col}}$ with $T_{\exp}$. The zoomed‐in inset shows an initial decrease of $T_{\mathrm{col}}$ with the increasing $T_{\exp}$.

As the rate of generation of excited triplet‐state PS(T_1_) is directly proportional to the concentration of PS,^[^ ^19^ ^]^ the disparity in rates of oxidation at inner and outer leaflets is a function of PS concentration difference $\Delta c=c_{\mathrm{in}}-c_{\mathrm{out}}$ ($c_{\mathrm{in}}$ and $c_{\mathrm{out}}$ are PS concentrations inside and outside of the vesicle, respectively) across the membrane. Therefore, to explore how increasing the contrast in oxidation rates at inner and outer leaflets would impact the vesicle stability and membrane rupture, we conducted experiments for four different PS concentration differences viz. Δc = 5, 10, 15 and 20 mM (see **Figure** **3**A, the error bars represents the standard error of a proportion^[^ ^44^ ^]^) for a continuous exposure of 80 s (see Table S1, Supporting Information for additional details). To observe appreciable fractions of affected vesicles, we used the higher concentrations of HPTS compared to that of methylene blue in the previous reported experiments.^[^ ^33^ ^]^ Such observations could be potentially attributed to lower singlet oxygen quantum yield and the weaker HPTS‐membrane interactions compared to methylene blue.^[^ ^34^, ^35^ ^]^ At a low Δc (=5 mM), we observed that the appearance of a series of transient pores was a frequent mode of membrane rupture and a large fraction (≈53%) of vesicles remained intact. However, upon increasing the exposure time up to 3 min, most vesicles (≈92%) opened transient pores. In contrast, at higher concentration differences Δc> 5 mM, none of the vesicles remained intact; the vesicles formed transient pores or exploded within 80 s. The total fraction of vesicles manifesting explosion (including both no‐curling and curling modes) increased with Δc (see Figure 3A). At Δc= 20 mM, ≈69% of vesicles showed an explosion, a sudden catastrophic collapse. To avoid any biases due to vesicle size distribution, we confirmed similar size distributions of vesicles (see Figure S4, Supporting Information) across all experiments. We as well note that the chamber surface used during illumination was hydrophobic. In our experiments, we did not observe any sign of vesicle attachment to the surface and therefore expect that the vesicle responses have no influence from the chamber surface. We also performed control experiments with Δc<0 and Δc=0 (see Figure S5, Supporting Information), and we observed that vesicle explosion or the formation of transient micron‐sized pores only occurs in cases of asymmetric distribution of PS (i.e., Δc≠0). Hence, the PS concentration difference impacts the membrane rupture, substantiating our argument that the asymmetric distribution of oxidation products could play a crucial role in determining the response of the lipid bilayer under light‐induced oxidation.

To further rationalize the influence of PS concentration difference on the dynamics of membrane rupture, we investigate its impact on two parameters: vesicle exposure time $T_{\exp}$, and vesicle collapse time $T_{\mathrm{col}}$. We define $T_{\exp}$ as the time duration of continuous irradiation until the membrane ruptures. The vesicle collapse time $T_{\mathrm{col}}$ is defined as the time duration from the moment when the membrane opens a pore till the vesicle completely flattens, and only applicable in cases of vesicle explosion. From the definition of $T_{\mathrm{col}}$, a higher value of $T_{\mathrm{col}}$ indicates a slower average pore opening speed. The vesicle exploding in the no‐curling mode predominantly have $T_{\mathrm{col}}⪅$ 100 ms, therefore, the pore opens faster in the no‐curling mode compared to the curling mode of vesicle explosion. Figure 3B shows the probability density of vesicles exploding in the no‐curling mode (i.e., $T_{\mathrm{col}}⪅$ 100 ms) increases with Δc. Furthermore, as demonstrated in Figure 3C as box plots,^[^ ^45^ ^]^ we observe a clear separation in $T_{\exp}$ for different modes of membrane rupture at all values of Δc (see Figure S6, Supporting Information for Δc= 5 mM with mostly transient pore formation). Vesicles collapsing in the no‐curling mode has the shortest $T_{\exp}$, followed by the vesicles exploding in the curling mode, while vesicles opening transients pores have the longest $T_{\exp}$. We note that the $T_{\exp}$ is only weakly correlated with the vesicles size (see Figure S7, Supporting Information). When Δc increases as shown in Figure 3C, $T_{\exp}$ for all modes of membrane rupture reduces, which signifies the overall increase in rate of lipid oxidation with increasing Δc. In Figure 3D, we plot the Gaussian‐weighted moving average of vesicle collapse time $T_{\mathrm{col}}$ with $T_{\exp}$ (see Figure S8, Supporting Information for individual vesicle data), which reveals a non‐monotonic nature of $T_{\mathrm{col}}$ of vesicle explosions for all Δc. Initially, as shown in the inset of Figure 3D, with the increasing $T_{\exp}$, $T_{\mathrm{col}}$ for vesicle collapse decreases. However, at longer $T_{\exp}$, $T_{\mathrm{col}}$ increases with the increasing $T_{\exp}$ of membrane rupture. In the later section, we will show how a non‐monotonic evolution of spontaneous curvature $H_{s}$ with $T_{\exp}$ could explain the non‐monotonic nature of $T_{\mathrm{col}}$.

### 2.4 Modeling Pore Dynamics of Vesicle Explosion

**Figure 4:**
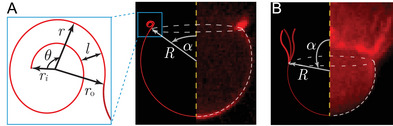
Model schematics of the A) curling, and B) no‐curling mode of vesicle explosion apposed to experimental images displaying the radius R and pore angle α of the spherical portion of the vesicle. In (A), the inset shows the rim of an opening pore modeled as a uniform spiral with a constant inter‐leaflet separation l. $r_{i}$ and $r_{o}$ are the inner and outer radius, respectively. r and θ are the radius and the angle as we move along the spiral. In B), tubule‐like structures sprout along the pore circumference.

To understand the impact of spontaneous curvature, $H_{s}$, induced by asymmetric oxidation on membrane stability, we develop a model for pore dynamics of vesicle explosion incorporating spontaneous curvature $H_{s}$. In the event of membrane rupture, vesicles only opened a single pore and largely maintained a spherical shape even in cases of vesicle explosion (see Figure 2C–E). Therefore, as depicted in **Figure** **4**A,B, we model the vesicle by an open sphere of radius R, and the pore angle α, subtended by the pore at the center. Upon membrane poration, the subsequent pore dynamics of a vesicle is governed by the competition between the release rate of total vesicle energy and the dissipation rate in the surrounding, formally known as Onsager's variational principle (for details, see Section S1, Supporting Information).^[^ ^46^ ^]^ This principle has successfully described the experimentally observed pore dynamics of vesicle rupture across contexts.^[^ ^32^, ^47^, ^48^, ^49^, ^50^, ^51^ ^]^ To model pore dynamics of vesicle explosion using Onsager's variational principle, in the following, we will develop the total vesicle energy E and the total Rayleigh dissipation potential Φ in the curling and no‐curling modes of vesicle explosion.

#### 2.4.1 Curling

The appearance and growth of bright spots observed at the pore edge in our experiments indicate accumulation of lipids around the pore rim (see Figure 2C). In the presence of $H_{s}$, the material accumulates around the pore rim as the pore edge curls up during the pore growth.^[^ ^47^, ^52^, ^53^, ^54^ ^]^ This curling occurs in the form of a spiral as observed in experiments^[^ ^53^, ^55^ ^]^ and simulations^[^ ^52^ ^]^ for red blood cell lysis and polymersomes bursting. Furthermore, the curling of the pore edge has been modeled in the form of a spiral to capture the corresponding pore dynamics.^[^ ^47^, ^53^, ^54^ ^]^ During vesicle explosion, we neglect the fusion of the lipid bilayer during curling, as the vesicle flattens in a short span of time (<500 ms), while the fusion of lipid bilayer is a slow stochastic event with a very large activation energy (occurring at a time scale of minutes to days).^[^ ^56^, ^57^ ^]^ In addition, prior works have shown that the incompatibility between positive lipid monolayer spontaneous curvature (ζ>0) and the membrane curvature during stalk formation restricts membrane fusion.^[^ ^58^ ^]^ Therefore the presence of TL in the oxidized membrane might further inhibits the fusion process during curling. Previous experimental and theoretical studies have suggested the membrane curling at the pore rim could be modeled as a uniform spiral.^[^ ^47^, ^53^, ^54^ ^]^ However, the current resolution of our experimental images limits our ability to demonstrate this phenomenon definitively. Further experimental efforts with improved image resolution are required to provide direct evidence for curling. Since we aim to bring forward a minimal model to capture pore dynamics of vesicle explosion, herein we model the lipid accumulation around the pore rim as a uniform spiral described by $r=r_{i}+l\theta /\left(2\pi \right)$ (Figure 4A). Here, $r_{i}$ and $r_{o}$ are the inner and outer radius of the spiral, respectively. l is uniform inter‐bilayer distance for each loop. r and θ are the radius and the angle as we move along the spiral. The pore opening in membrane reduces the membrane area strain, and thereby relaxing the membrane tension.^[^ ^32^, ^50^, ^51^ ^]^ Therefore, in cases of vesicle explosion, we assume the membrane stretching energy to be negligible beyond the initial pore opening angle $\alpha_{0}$ measured in the first experimental image showing the pore. Thus, we express the total energy of a vesicle in the curling mode of explosion as

(1) $$E_{c}=E_{p}+E_{b}^{\mathrm{sph}}+E_{b}^{\mathrm{rim}}$$

including the pore edge energy $E_{p}$ (Equation S3, Supporting Information), and the bending energy of the spherical membrane section $E_{b}^{\mathrm{sph}}$ (Equation S5, Supporting Information) and the pore rim of the vesicle $E_{b}^{\mathrm{rim}}$ (Equation S6, see Section S1A, Supporting Information for detailed derivation).

Meanwhile, the total dissipation potential in the curling model of vesicle explosion is written as (see Section S1B, Supporting Information for detailed derivation)

(2) $$\Phi_{c}=\;\Phi_{a}+\;\Phi_{r}+\;\Phi_{\mathrm{sm}}+\;\Phi_{\mathrm{rm}}$$

Here, $\Phi_{a}$ describes the viscous losses in the aqueous solution from the flow field in the vicinity of the membrane as the pore expands (Equation S14, Supporting Information). $\Phi_{r}$ refers to the viscous dissipation arising in the aqueous solution due to the pore rim retracting, which is modeled as a cylinder moving in a viscous fluid (Equation S15, Supporting Information). Additionally, the viscous losses occurs due to the flow of lipids within the spherical section of the membrane and into the rim,^[^ ^54^ ^]^ as $\Phi_{\mathrm{sm}}$ and $\Phi_{\text{rm}}$ (Equations S22 and S23, Supporting Information), respectively.

#### 2.4.2 No‐Curling

In contrast to the curling mode of the explosion, we did not observe material accumulation around the pore rim in the no‐curling mode. Instead, a tubule‐like structure sprouts from the rim as shown in Figure 4B, similar to tubule growth in vesicles destabilized by electroporation.^[^ ^43^, ^59^ ^]^ During the electroporation, the spontaneous curvature was generated due to asymmetry in the lipid bilayer caused by charged lipids^[^ ^43^ ^]^ or by asymmetric adsorption of conical glycolipid.^[^ ^59^ ^]^ In our experiments we observe similar bursting of vesicles with no lipid accumulation around the edge and consider the spontaneous curvature generated by asymmetric oxidation (see Figure 2B). Therefore, for no‐curling explosion we consider the sprouting of tubules from the pore rim as a minimal model for bending energy of the vesicle. It has been further shown that these tubular structure originating from the pore rim significantly reduces the line tension of the lipid bilayer.^[^ ^59^ ^]^ Therefore, we neglect the pore edge energy and consider only the bending energy of the spherical membrane section in the total energy, as

(3) $$E_{\mathrm{nc}}=E_{b}^{\mathrm{sph}}$$

In the absence of material accumulation around the rim as in the no‐curling mode of explosion, the total dissipation potential consists of dissipation in the surrounding solution and the spherical section of the membrane, given as

(4) $$\Phi_{\mathrm{nc}}=\Phi_{a}+\Phi_{\mathrm{sm}}$$

#### 2.4.3 Model Predictions

**Figure 5:**
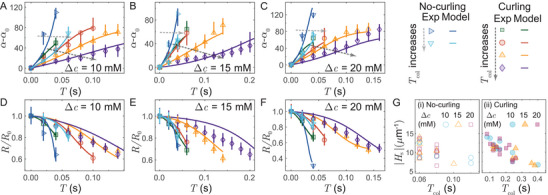
Model prediction for pore dynamics of vesicle explosion. A–C) Experimentally observed and model predicted profiles of the pore angle α for a total of six exploding vesicles (two showing the no‐curling mode, and four showing the curling mode) at Δc= 10, 15, and 20 mM. The dotted arrows show the direction of increasing $T_{\mathrm{col}}$. D–F) Experimentally observed and model predicted profiles of the vesicles radius R for the corresponding vesicles. $\alpha_{0}$, and $R_{0}$ are the pore angle and vesicle radius measured in the first image showing the pore in each experiment. G) The magnitude of the spontaneous curvature $|H_{s}|$ decreases as $T_{\mathrm{col}}$ increases for the i) no‐curling and ii) curling modes of vesicle explosion.

We apply Onsager's variational principle to Equations ((1), (2)), and Equations ((3), (4)), to obtain the governing equations for evolution of the pore angle α and the vesicle radius R in the curling and no‐curling modes of vesicle explosion, respectively. The spontaneous curvature $H_{s}$ remains the only unknown parameter, which we determine by fitting the pore profiles obtained from experiments to model prediction (see Section S1, Supporting Information for details). In **Figure** **5**, we show a comparison of model predictions and experimentally obtained profiles of pore angle α (Figure 5A–C) and vesicle radius R (Figure 5D–F) for six vesicles (two no‐curling, and four curling cases) per concentration difference of PS (Δc= 10, 15, and 20 mM). $\alpha_{0}$, and $R_{0}$ are the pore angle and vesicle radius at the first experimental image frame at which a pore is observed. As evidenced from Figure 5A–F, our model captures the pore dynamics of vesicle explosion reasonably well. In the curling mode of vesicle explosion, our model captures the evolution of pore angle α up to an angle of ≈120° and vesicle radius R up to $\approx \;\;R_{0}/2$, beyond which the assumption $r_{o}\ll R\sin\alpha$ in deriving the bending energy of the pore rim $E_{b}^{\mathrm{rim}}$ (see Equation S6, and Section S1A, Supporting Information)) fails. In the no‐curling mode, no such assumption is required and the model can capture the vesicle evolution for even wider ranges.

Specifically, we demonstrate that the spontaneous curvature $H_{s}$ plays a dominant role in controlling the pore dynamics of vesicle explosion. After the membrane ruptures, the vesicle radius *R* shrinks as the inner content of vesicle leaks through the pore, and the pore angle α grows. As shown in Figure 5G, with the increase in the vesicle collapse time $T_{\mathrm{col}}$ (i.e., as the pore opens slower, for instance, in the direction of the dotted arrow in Figure 5A–C), the magnitude of the induced spontaneous curvature $|H_{s}|$ decreases, for both the no‐curling and curling modes of vesicle explosion. Our results suggest that the spontaneous curvature $H_{s}$ determines the rate of pore angle α expansion as well as the shrinking rate of vesicle radius R since it controls the rate of release of total energy as the pore expands.

## 3 Discussion

### 3.1 Non‐Monotonic Temporal Evolution of Spontaneous Curvature

To shed light on the oxidation kinetics of the lipid membrane and the manner in which asymmetric oxidation generates spontaneous curvature, here we relate the asymmetry in TL distribution across the inner and outer leaflets with the corresponding induced $H_{s}$. As noted earlier, the lipid oxidation is considered as a two‐step consecutive reaction following first‐order kinetics: LH $\overset{k^{\prime }}{\to }$ LOOH $\overset{k}{\to }$ TL.^[^ ^18^ ^]^ However, since $k^{\prime }\gg k$, we only consider the second step in our further analysis. The impermeability of PS to the lipid membrane and its proximity to the inner leaflet leads to faster oxidation of the inner leaflet compared to that of the outer leaflet, which results in asymmetric distribution of TL in the lipid bilayer (Figure 2A). Thus, we choose different reaction rate constants, $k_{\mathrm{in}}$ and $k_{\mathrm{out}}$, for reaction at the inner and outer leaflets, respectively. Accordingly, we can write the difference of mole fraction of TL, Δϕ, as

(5) $$\Delta \phi =\phi_{\mathrm{out}}-\phi_{\mathrm{in}}=e^{-k_{\mathrm{in}}t}-e^{-k_{\mathrm{out}}t}$$

where $\phi_{\mathrm{out}}$ and $\phi_{\mathrm{in}}$ are the mole fractions of the TL in the outer and inner leaflet, respectively. The composition of the lipid constituents determines the spontaneous curvature of a uniformly mixed multicomponent monolayer considering the linear additivity.^[^ ^60^, ^61^ ^]^ Therefore, the spontaneous curvature of inner and outer leaflets evolve with the accumulation of TL as the oxidation progresses. Consequently, following prior works,^[^ ^60^, ^61^ ^]^ we write the spontaneous curvature of the outer leaflet $H_{s}^{\mathrm{out}}=\zeta \phi_{\mathrm{out}}$, and inner leaflet $H_{s}^{\mathrm{in}}=\zeta \phi_{\mathrm{in}}$. Here, ζ represents the effective monolayer spontaneous curvature of TL. Furthermore, the induced bilayer spontaneous curvature $H_{s}=\left(H_{s}^{\mathrm{out}}-H_{s}^{\mathrm{in}}\right)/2$ considering the bending rigidity of both outer and inner leaflets to be equal, $k_{b}^{\mathrm{out}}\approx k_{b}^{\mathrm{in}}=k_{b}$.^[^ ^62^ ^]^ Using Equation (5), we relate the induced $H_{s}$ with Δϕ as

(6) $$H_{s}=\frac{1}{2}\zeta \Delta \phi =\frac{1}{2}\zeta \left(e^{-k_{\mathrm{in}}t}-e^{-k_{\mathrm{out}}t}\right)$$

**Figure 6:**
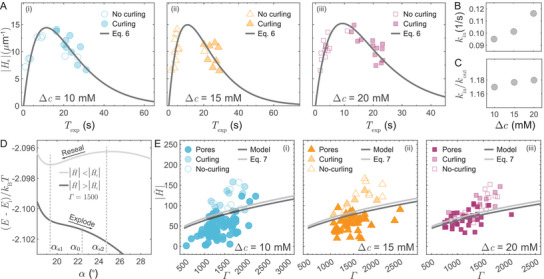
Characterization of the temporal evolution of spontaneous curvature, light‐induced asymmetric oxidation, and phase diagram for curvature‐assisted vesicle explosion. A) A non‐monotonic generation of spontaneous curvature due to lipid bilayer asymmetric oxidation over time, B) Linear increase in rate of oxidation $k_{\mathrm{in}}$ at inner leaflet with Δc. C) Ratio of oxidation rate at inner leaflet and outer leaflet $k_{\mathrm{in}}/k_{\mathrm{out}}$ at different Δc. D) Modulation of the energy landscape by non‐dimensional spontaneous curvature $\tilde{H}$ with growing pore angle α at a representative non‐dimensional line tension Γ= 1500. $E_{i}$ is the total energy of the vesicle just before the membrane ruptures. $\alpha_{s1}$, and $\alpha_{s2}$ are the two stationary points when $\tilde{H}<{\tilde{H}}_{c}$. E) Phase diagram for vesicle explosion and pore formation in the parameter space of $\tilde{H}$ and Γ for different PS concentration differences across the membrane.

As shown in **Figure** **6**A, we plot the fitting curve of Equation (6) to the experimentally acquired $H_{s}$, and further infer the oxidation rates $k_{\mathrm{out}}$ and $k_{\mathrm{in}}$ (Figure 6B,C). We choose ζ= 0.5 nm^−1^ as the effective monolayer spontaneous curvature of TL.^[^ ^29^, ^61^, ^63^, ^64^ ^]^ Equation (6) explicates the non‐monotonic temporal evolution of experimentally obtained spontaneous curvature $H_{s}$. Initially, the asymmetric distribution Δϕ of TL increases, due to differential rates of oxidation at inner and outer leaflets, i.e., $k_{\mathrm{in}}\neq k_{\mathrm{out}}$, and therefore the magnitude of spontaneous curvature $|H_{s}|$ increases. However, as the oxidation progresses toward completion, Δϕ decays along with $|H_{s}|$. Our results infer a maximum mole fraction difference |Δϕ|≈6% of TL across the leaflets. The values of the spontaneous curvature $H_{s}$ (in the range of 5 − 15 µm^−1^) obtained in the current study are similar to earlier measurements.^[^ ^42^ ^]^ Using optical tweezers to measure the force required to pull tubes from GUVs, Dasgupta et al.,^[^ ^42^ ^]^ obtained the spontaneous curvatures of ≈5 µm^−1^, generated by a mole fraction difference of ≈ 4% of a conical glycolipid (GM1) between the inner and outer leaflets. In addition, since PS is encapsulated inside the vesicle, we obtain $k_{\mathrm{in}}>k_{\mathrm{out}}$ with an average asymmetry $k_{\mathrm{in}}/k_{\mathrm{out}}-1=18\pm 0.5\%$ in oxidation rates across all concentration differences Δc of PS (Figure 6C). We note that in cases of PS outside only (Δc<0), since the contact‐dependent reaction occurs faster at the outer leaflet, i.e., $k_{\mathrm{out}}>k_{\mathrm{in}}$, the spontaneous curvature generated would be opposite to the cases of PS inside only (Δc>0). However, as the vesicle bending energy is proportional to the square of the deviation of the instantaneous membrane curvature from the spontaneous curvature (see Equation S5, Supporting Information), there will be an energetic penalty even in cases of PS outside only (Δc<0) causing vesicle disruption as observed in our experiments (see Figure S5C, Supporting Information).

### 3.2 Spontaneous Curvature Assists Vesicle Explosion

To highlight the role of the spontaneous curvature $H_{s}$, in Figure 6D we show general impact of non‐dimensional spontaneous curvature $\tilde{H}=H_{s}R_{0}$ on the total energy $\left(E-E_{i}\right)/k_{B}T$ including stretching, pore‐edge and bending energy of a vesicle (see Section S2A, Supporting Information for details). $E_{i}$ is initial energy of a vesicle moment before a pore opens. Here, we consider a representative non‐dimensional line tension $\Gamma =\gamma R_{0}/k_{b}=$ 1500 in our experiments, where γ is the line tension of lipid bilayer. We note that before the pore opening, the induced $H_{s}$ causes budding off of smaller vesicles or tubule‐like structures (see Movies S2 and S3, Supporting Information), reducing the membrane area and thus effectively stretching the membrane. The resulted membrane tension ultimately causes the membrane to rupture. As shown in Figure 6D, the pore opens quickly up to $\alpha_{s1}$ (given by $\partial E/\partial \alpha =0,\partial^{2}E/\partial \alpha^{2}>0$), where the membrane tension is relaxed σ≈0. In the case of $|\tilde{H}\left|<\right|{\tilde{H}}_{c}|$, the energy profile has a second stationary point $\alpha_{s2}$ (given by $\partial E/\partial \alpha =0,\partial^{2}E/\partial \alpha^{2}<0$). Beyond $\alpha_{s1}$ only pore edge energy $E_{p}$ and the bending energy $E_{b}$ remains to compete. While after $\alpha_{s2}$, the bending energy dominates and creates a favorable energy gradient for the pore to grow. However, for $|\tilde{H}\left|<\right|{\tilde{H}}_{c}|$, there is an energy barrier between the stationary points $\alpha_{s1}$ and $\alpha_{s2}$. After the pore expands to $\alpha_{s1}$, the pore edge energy $E_{p}$ resists any further expansion and reseals the pore. On the other hand, for $|\tilde{H}\left|>\right|{\tilde{H}}_{c}|$ (see Figure 6D), the energy barrier vanishes. Hence, in case of $|\tilde{H}\left|>\right|{\tilde{H}}_{c}|$, beyond $\alpha_{0}\approx \cos^{-1}\left(2/\left(1+\varepsilon_{0}\right)-1\right)$ (where σ≈0), the pore encounters a favorable energy gradient and keeps growing, resulting into vesicle explosion (see Section S2A, Supporting Information for details). We consider the critical area strain $\varepsilon_{0}=4\%$ at which the membrane ruptures, consistent with the experimentally observed values for photosensitized DOPC vesicle rupture.^[^ ^25^ ^]^ The later pore dynamics is governed by the competition between the bending energy and pore edge energy. Considering a favorable energy gradient ${\partial E/\partial \alpha |}_{R_{0},\alpha_{0}}<0$, we obtain the critical spontaneous curvature as

(7) $$|{\tilde{H}}_{c}|\approx \sqrt{2\Gamma \cot\alpha_{0}}$$

for both no‐curling and curling modes of vesicle explosion (see Section S2B,C, Supporting Information for details). As shown in Figure 6D, when $|\tilde{H}\left|<\right|{\tilde{H}}_{c}|$, the pore edge energy dominates, creating an adverse energy gradient that forces the pore to reseal and thereby instantiating transient pore formation (Figure 2D). However, when $|\tilde{H}\left|>\right|{\tilde{H}}_{c}|$ the bending energy dominates, favoring the pore to expand further and thus resulting in the vesicle explosion (Figure 2B,C). Therefore, the spontaneous curvature $H_{s}$ generated by light‐induced asymmetric oxidation assists in destabilizing vesicles and even explosion by modulating the energy gradient.

To further delineate how the competition between the bending energy and the pore edge energy determines the mode of the vesicle rupture, we present a phase diagram considering the $\tilde{H}-\Gamma$ parameter space as shown in Figure 6E. To compute $\tilde{H}=H_{s}R_{0}$ and $\Gamma =\gamma R_{0}/k_{b}$ for all experimental data, we estimate the vesicle radius $R_{0}$ just before the moment when the membrane ruptures. Furthermore, for the cases of the no‐curling and curling modes of vesicle explosion, we use $H_{s}$ obtained from the pore‐opening dynamics. While in the cases of transient pore formation, we compute $H_{s}$ using Equation (6) at the moment of rupture. The solid dark boundary between the vesicle explosion and transient pore formation in Figure 6E is determined by performing numerical experiments to obtain the lowest value of $\tilde{H}$ at a given Γ leading to vesicle explosion. On the other hand, Equation (7) gives the analytically predicted boundary, as the light gray line in Figure 6E. Both numerically and analytically obtained boundaries delineate the experimentally observed vesicle explosion and transient pore formation reasonably well, highlighting the crucial role of spontaneous curvature $\tilde{H}$ in the non‐equilibrium response of vesicles.

Notably, the present framework also aligns well with several other experimental observations. In our experiments with the asymmetric oxidation, the overall light‐induced oxidation rate increases with the encapsulated PS concentration (see Figure 6B), and thus $T_{\exp}$ across all modes of membrane rupture decreases as shown in Figure 3C. Moreover, $H_{s}$ tends to decay at larger $T_{\exp}$, in which the pore edge energy dominates for transient pore formation. This is consistent with our observation that the vesicles tend to open transient pores at longer $T_{\exp}$ (Figure 3C). As noted earlier, the spontaneous curvature $H_{s}$ regulates the pore expansion rate (Figure 5). In line with this view, due to initial increase in $H_{s}$ at shorter $T_{\exp}$, $T_{\mathrm{col}}$ first decreases (i.e., pore opens faster) with the increasing $T_{\exp}$ (inset of Figure 3D). However, at longer $T_{\exp}$ when $H_{s}$ decays, the experimentally observed rate of pore expansion is slower and thus $T_{\mathrm{col}}$ increases with longer $T_{\exp}$, as shown in Figure 3D.

## 4 Conclusion

In conclusion, we investigate the crucial role for the dramatic conformational changes occurring during lipid oxidation on the integrity and stability of lipid vesicles. Specifically, we link the changes in molecular conformation to the generation of spontaneous curvature. Our model captures experimentally observed pore dynamics and enables estimates of spontaneous curvature as the pore grows. We further connect the generated spontaneous curvature with oxidation kinetics of the lipid membrane, and rationalize the temporal evolution of experimentally derived spontaneous curvature.

By estimating the reaction rate constants, we reveal that oxidation reactions at the inner leaflet occur ≈18% faster than the outer leaflet when the photosensitizer is encapsulated within vesicles in current experiments, leading to a ≈6% difference in mole fraction for oxidation product distribution across leaflets. Even for such a small asymmetry in oxidation product distribution, the introduced spontaneous curvature is sufficient to cause the vesicle explosion. Therefore, in biological membranes, even a small asymmetric distribution of cholesterol and unsaturated lipids could ultimately be crucial in determining biological membrane integrity under asymmetric oxidation.

Moreover, this work shows how induced spontaneous curvature modulates the energy landscape of pore expansion within a vesicle and assists in driving vesicles to explosion. We provide a phase diagram and analytical criteria to predict the fate of vesicles in forming a transient pore or proceeding to explosion. Our work could not only aid in strategizing the deliberate destabilization of membranes in the emerging field of photodynamic therapy, but also help in the development of remotely triggered release for vesicle‐based drug delivery system. We remark that while we rationalize our observed results based on the hypothesis of asymmetric distribution of oxidized lipids as a plausible mechanism, alternative mechanisms–such as the removal of oxidized lipids discussed in previous works ^[^ ^25^, ^65^ ^]^‐may also account for the experimental observations. Future theoretical models based on these alternative mechanisms and further experimental investigations are necessary to evaluate the different hypotheses.

Our work may also provide an explanatory mechanism for the well established observation that membrane‐bound cholesterol protects against oxidative damage.^[^ ^23^, ^27^ ^]^ Cholesterol can increase the ordering of lipid tails and tight molecular packing.^[^ ^23^ ^]^ In the presence of truncated lipid tails produced from the oxidation process, cholesterol acts to prevent the truncated lipid chain from reorienting toward the bilayer‐water interface.^[^ ^66^ ^]^ This prevents further shape deformation of lipids, and thereby reduces the effective monolayer spontaneous curvature ζ of TL. Such a decrease of the membrane spontaneous curvature generated by oxidation may ultimately protect the membrane integrity. We believe that this insight would imply that asymmetric distribution of cholesterol may influence the change of the effective monolayer spontaneous curvature ζ of TL and the oxidation rates differentially between the inner and outer leaflets. Future research will be required to clarify the role of bilayer molecular asymmetry in buffering or enhancing damage incurred from oxidative assault.

## 5 Experimental Section

### Materials

1,2‐dioleoyl‐sn‐glycero‐3‐phosphocholine (DOPC) for GUVs fabrication and 1,2‐dipalmitoyl‐sn‐glycero‐3‐phosphoethanolamine‐N‐(lissamine rhodamine B sulfonyl) (ammonium salt) (Rh‐DPPE) to label the GUVs were purchased from Avanti Polar Lipids. Other chemicals, including 8‐Hydroxypyrene‐1,3,6‐trisulfonic acid trisodium salt (HPTS) used as photosensitizer, organic solvent paraffin oil, and sugars (sucrose and glucose) were purchased from Sigma–Aldrich (US). The triplet quantum yield of HPTS was $\Phi_{T}\approx 1-\Phi_{F}\approx$ 0.18 assuming other nonradiative decay rates negligible, where fluorescence quantum yield of HPTS was $\Phi_{F}=$ 0.82 in aqueous solutions as shown in previous work.^[^ ^67^ ^]^ Since the production of singlet oxygen was primarily dependent on the generation of triplet states,^[^ ^68^ ^]^ it was estimated that the singlet quantum yield of HPTS was $\Phi_{\Delta }\leq \Phi_{T}\approx 0.18$. The electronic absorbance spectrum of a 100 µM HPTS in 500 mM sucrose solution is shown in Figure S9 (Supporting Information).

### Lipids‐in‐Oil Solution

To prepare lipids‐in‐oil solution, DOPC (25 mg mL^−1^) and Rh‐DPPE (1 mg mL^−1^) stock solutions solubilized in chloroform were used. Briefly, a thin film of dried lipids (32 µL of DOPC and 13 µL of Rh‐DPPE) was formed in a clean glass vial by evaporating the chloroform under a gentle airflow. Afterward, to further remove the traces of the chloroform, the vial was kept in a desiccator overnight. A 5 mL of paraffin oil was added to the glass vial to give a final concentration of lipids ≈200 µM DOPC and 2 µM Rh‐DPPE. This was followed by sonication for 30 min. Finally, the solution was incubated for 24 h at room temperature to ensure the complete dissolution of the lipids. After incubation, the lipid‐in‐oil solution was stored at room temperature and used to fabricate GUVs for upto four days.

### Inner and Outer Solutions

GUVs were fabricated encapsulating 500 mM Sucrose and 5/10/15/20 mM HPTS. To ensure an isomolar outer solution with the inner solution, the outer solution with glucose concentrations of 505/510/515/520 mM was used. A concentration of 500 mM sucrose/glucose was chosen for inner and outer solutions, respectively, to improve the vesicle yield by increasing the density gradient and facilitating the transfer of emulsion droplets through the LOS‐glucose interface.^[^ ^69^ ^]^ All aqueous solutions were made using DI water.

### Inverted Emulsion Method

Initially, 150 µL of glucose (outer) solution was added to a 2 mL centrifuge tube. This was followed by a gentle layering of 50 µL if lipid‐in‐oil solution on top of glucose solution. To form an interfacial lipid monolayer, the entire set‐up was incubated for 30 min. Afterward, in a different centrifuge tube, 10 µL of sucrose and HPTS (inner) solution was added to a 250 µL of lipid‐in‐oil solution. To yield a water‐in‐oil emulsion, this tube was mechanically agitated on a vortex mixture (3000 RPM) for a minute. Immediately after, 100 µL of this emulsion was gently layered on the top of the interface and centrifuged at 200 g for 3 min. A 100 µL of GUVs solution was extracted from the bottom 150 µL of glucose solution. For further visualization, an aliquot of 10 µL GUVs dispersion was diluted in a 100 µL isotonic glucose solution.

### Confocal Microscopy Visualization and Irradiation

Confocal microscopy images were acquired on an inverted Zeiss LSM 7 Live confocal line‐scanning microscope equipped with an EC Plan‐Neofluar 20× /0.50 objective in fluorescence mode. Samples (110 µL) were injected in a 18‐well chamber (μ‐Slide 18 Well, ibidi) that was covered to prevent evaporation. The two channel image collection was set up as follows: HPTS was excited with a diode laser at 405 nm (2.82 mW at 100% power) with emission detected in the wavelength range of 415–455 nm, while the rhodamine‐based lipid label was excited at 561 nm (306 µW at 16% power) with emission detected in the wavelength range of 575–600 nm. Images were collected at a rate of 50 frames per sec and a resolution of 256 × 256 pixels. Further analysis of fluorescence confocal acquisitions was performed with the ImageJ software.

## Author Contributions

V.K.M., O.S.P., and J.F. designed the research. V.K.M. performed the experiments and numerical simulations. V.K.M. and J.F. analyzed the data. V.K.M., O.S.P., and J.F. wrote the manuscript.

## Conflict of Interest

The authors declare no conflict of interest.

## Supporting information

Supporting Information

Supplemental Movie 1

Supplemental Movie 2

Supplemental Movie 3

## Data Availability

The data that support the findings of this study are available from the corresponding author upon reasonable request.
