# Supplementary material for: Curvature‐Assisted Vesicle Explosion Under Light‐Induced Asymmetric Oxidation
Source: Adv Sci (Weinh). 2024 Aug 13;11(38):2400504. doi: 10.1002/advs.202400504 (PMC11481189; doi:10.1002/advs.202400504)
Supplement: Supplementary file 1 — Supporting Information [file ADVS-11-2400504-s003.pdf]

ADVANCED SCIENCE
Open Access

Supporting Information

for *Adv. Sci.*, DOI 10.1002/advs.202400504

Curvature-Assisted Vesicle Explosion Under Light-Induced Asymmetric Oxidation

*Vinit Kumar Malik, On Shun Pak and Jie Feng**

Supplementary Information for

Curvature-assisted vesicle explosion under light-induced asymmetric oxidation

Vinit Kumar Malik, On Shun Pak, Jie Feng

Jie Feng
E-mail: jiefeng@illinois.edu

This PDF file includes:

Supplementary Text
Figs. S1 to S9
Table S1
Captions for Movies S1 to S3
SI References

Other supporting materials for this manuscript include the following:

Movies S1 to S3

Supplementary Text

1. Modeling pore dynamics of vesicle explosion

To develop the governing equation for pore dynamics in a vesicle explosion, we model the vesicle, as shown in Fig. S1(A-B), by an open sphere of radius R, and the pore angle α. Once the pore opens, following Onsager's variational principle (1), pore growth is controlled by the competition between rates of energy release and dissipation. This variational principle has successfully described the experimentally observed pore dynamics of vesicle membrane rupture (2–7), and is written as

$$\nabla E_{\mathrm{i}}^{\mathrm{sph}} = -\nabla_{\mathrm{u}} \Phi_{\mathrm{i}} \tag{S1}$$

where $\nabla = \left(\partial/\partial R, \partial/R\partial\alpha\right)$, and $\nabla_{\mathrm{u}} = \left(\partial/\partial\dot{R}, \partial/\partial\left(R\dot{\alpha}\right)\right)$. Here, $\dot{R}$ represents rate of vesicle radius shrinkage, and $R\dot{\alpha}$, characterizes the rate of pore expansion. E_{i}, and Φ_{i} represents the total energy, and the total dissipation potential function of the system, respectively; $\mathrm{i} = \mathrm{c}$ or nc for curling or no-curling mode of vesicle explosion. In the rest of this section, we will derive equations for the total energy E and the total dissipation potential Φ.

A. Energy of a vesicle. A classical continuum form of the total energy of a vesicle with an open pore, $E = E_{\mathrm{s}} + E_{\mathrm{p}}$ (2, 4), comprises of energy contributions due to membrane stretching energy

$$E_{\mathrm{s}} = \sigma^2 A_0/2K, \tag{S2}$$

and pore edge energy

$$E_{\mathrm{p}} = 2\pi\gamma R \sin\alpha. \tag{S3}$$

Here, σ, K, and γ are the membrane tension, elastic stretching modulus and line tension of lipid membrane, respectively, whereas A_0 is the surface area of the unstretched vesicle. Prior experimental studies suggest the disruption in oxidation-induced lipid packing and generation of spontaneous curvature, driving budding of small vesicles and tubule-like structures from the vesicle membrane (8–10). Aligned with this view, we observe the tubule-like structures budding off the vesicles in our experiments (see SI movies S1 and S2). The loss in membrane area from these budding events causes membrane area strain, resulting in the build up of membrane tension σ. Once the critical membrane tension is achieved, membrane ruptures and opens a pore. The relaxation of membrane stretching energy E_{s} drives the initial pore growth. The pore often grows quickly (within a few ms) to reduce the membrane area strain, and thereby relaxing the membrane tension *i.e.* $\sigma \approx 0$ (2, 6, 7). Therefore, in cases of vesicle explosion, we assume the membrane stretching energy to be negligible beyond the initial pore opening angle α_0 (at the first image frame where the pore is observed).

In our previous studies for osmotic-induced rupture of vesicles, we showed the critical role played by the bending energy $E_{\mathrm{b}} = \frac{1}{2}k_{\mathrm{b}}\int_A (H - H_{\mathrm{s}})^2\,\mathrm{d}A$ in unifying the experimentally observed different pore dynamics (6, 7). Here, k_{b} is the bending rigidity of a lipid bilayer, A denotes the instantaneous surface area of a vesicle, and H represents the instantaneous local curvature of the vesicle. Thus in cases of vesicle explosion where the asymmetric oxidation generates spontaneous curvature H_{s}, beyond the pore angle α_0 the total energy includes pore edge energy and bending energy given as

$$E = E_{\mathrm{p}} + E_{\mathrm{b}} = 2\pi\gamma R\,\sin\,\alpha + \frac{1}{2}k_{\mathrm{b}}\int_A (H - H_{\mathrm{s}})^2\,\mathrm{d}A. \tag{S4}$$

Total energy in curling mode of vesicle explosion. For vesicle explosion in the curling mode, we observe the lipid accumulation around the pore rim, which we describe in form of a uniform spiral of radius $r = r_{\mathrm{i}} + \frac{l}{2\pi}\theta$, where r_{i}, l, and θ are the inner radius of the spiral, inter-bilayer separation of spiral loop, and angle subtended at center as we move along the spiral, respectively (see Fig. 4 of main text). Therefore, inspired by previous studies (3, 11–13) and to bring forward a minimal model to describe pore dynamics of vesicle explosion, we model the lipid accumulation around the pore rim in a form of uniform spiral described by $r = r_{\mathrm{i}} + \frac{l}{2\pi}\theta$ (Fig. 4A). Here, r_i and l are the inner radius and uniform inter bilayer distance for each loop, respectively. r, and θ are defined as the radius and angle from the inner radius r_{i}, as we traverse along the spiral. At the open pore edge, the moment-free boundary condition dictates $r_i = |H_{\mathrm{s}}^{-1}|$ (14). We choose $l = 10$ nm, similar to the typical values in the modeling for edge curling of red blood cell lysis, as well as the inter bilayer separation in a stack of bilayers due to steric and van der Wall interactions (11, 13, 15). Therefore, the bending energy E_{b} in Eq. S4 could be split into the bending energy contribution from the spherical section

$$E_{\mathrm{b}}^{\mathrm{sph}} = \frac{1}{2}k_b\left(H - H_{\mathrm{s}}\right)^2 A_{\mathrm{sph}}, \tag{S5}$$

and the pore rim

$$\begin{aligned} E_{\mathrm{b}}^{\mathrm{rim}} &= \frac{1}{2}k_{\mathrm{b}}\int_{A_{\mathrm{rim}}}\left(\frac{1}{r} - |H_{\mathrm{s}}|\right)^2\mathrm{d}A \\ &= \frac{1}{2}k_b\int_{r_i}^{r_o}\left(\frac{1}{r^2} - 2\frac{|H_{\mathrm{s}}|}{r} + H_{\mathrm{s}}^2\right)2\pi R\sin\alpha\frac{2\pi}{l}r\ \mathrm{d}r \\ E_{\mathrm{b}}^{\mathrm{rim}} &= \frac{1}{2}k_{\mathrm{b}}\left(\frac{2}{r_{\mathrm{o}}^2 - r_{\mathrm{i}}^2}\left(\ln\left(\frac{r_{\mathrm{o}}}{r_{\mathrm{i}}}\right) - 2|H_{\mathrm{s}}|\left(r_{\mathrm{o}} - r_{\mathrm{i}}\right)\right) + H_{\mathrm{s}}^2\right)A_{\mathrm{rim}} \end{aligned} \tag{S6}$$

Here, $A_{\mathrm{sph}} = 2\pi R^2(1+\cos\alpha)$ is the membrane area of spherical section, and $A_{\mathrm{rim}} = 2\pi^2 R\sin\alpha(r_{\mathrm{o}}^2 - r_{\mathrm{i}}^2)/l$ is the membrane area of curled pore edge around the rim. In writing Eq. S6, we consider the local curvature of pore rim $H = \frac{1}{r} + \frac{1}{R\sin\alpha} \approx \frac{1}{r}$ as $R\sin\alpha >> r$.. We note that Eq. S6 is consistent with the expression for the bending energy of the curled pore edge as obtained by Callan *et. al.* (13). Given the short T_{col} of only few hundreds of milliseconds, we consider the membrane material loss to be negligible. Therefore, we consider the total area $A = A_{\mathrm{sph}} + A_{\mathrm{rim}} = 2\pi R_0^2(1+\cos\alpha_0)$ to be conserved and compute the outer radius $r_{\mathrm{o}} = \sqrt{(A - A_{\mathrm{sph}})l/(2\pi^2 R\sin\alpha) + r_{\mathrm{i}}^2}$. Finally, following Eqs. S4, S5, and S6, the total energy of a vesicle in cases of vesicle explosion in the curling mode is given by

$$E_{\mathrm{c}} = E_{\mathrm{p}} + E_{\mathrm{b}}^{\mathrm{sph}} + E_{\mathrm{b}}^{\mathrm{rim}}. \tag{S7}$$

Total energy in curling mode of vesicle explosion. In the no-curling mode of explosion, a network of tubules sprouted from the rim as shown in Fig. S1B, also seen in vesicle destabilization by electroporation (16, 17). In the presence of these tubular structures, the line tension of the lipid bilayer is significantly reduced (17). Therefore, total energy of vesicle explosion in the no-curling mode would have contribution only from the bending energy of spherical section given as

$$E_{\mathrm{nc}} = E_{\mathrm{b}}^{\mathrm{sph}}. \tag{S8}$$

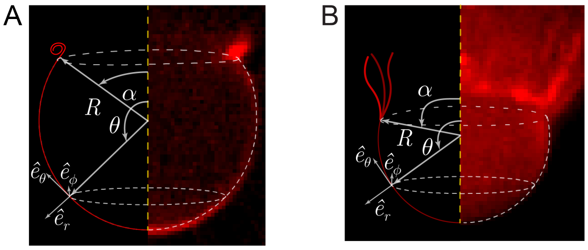

Fig. S1. Model schematics of *(A)* curling, and *(B)* no-curling mode of vesicle explosion apposed to experimental images displaying the radius R and pore angle α of the spherical portion of the vesicle.

B. Dissipation of energy during pore expansion.

Dissipation potential of viscous losses in the aqueous solution. The relaxation of the stored elastic energy, as the pore grows, sets the surrounding solvent fluid in motion. Hence, viscous losses in the solvent, provides a route to dissipate elastic energy stored in the vesicle membrane (18, 19). Following prior works (5, 18, 19), to account for the viscous damping in aqueous solution during the pore life considering the low Reynolds number flow, we write the Rayleigh dissipation function as

$$\varPhi_{\mathrm{a}} = -2\pi R^2 \Delta p_1 \dot{R} + \eta_{\mathrm{s}} R\left(C_{\mathrm{a2}}\dot{R}\,(R\dot{\alpha}) + \frac{1}{3}C_{\mathrm{a2}}\tan\frac{\alpha}{2}\,(R\dot{\alpha})^2\right). \tag{S9}$$

Here, $\dot{R}$ represents rate of vesicle radius shrinkage, and $R\dot{\alpha}$, characterizes the rate of pore expansion. η_s is the solvent viscosity. The resistance coefficient $C_{\mathrm{a2}} = 12((\pi - \alpha - \sin\alpha)\tan\frac{\alpha}{2} + (\pi-\alpha)^2)$. The pressure difference Δp_1 across membrane is generated due to the flow caused by vesicle radius shrinkage or expansion. Since, during explosion, the vesicle did not move along the axis of symmetry (yellow dotted line on Fig. 4 (A-B) of main text), in writing Eq. S9, we neglect the viscous dissipation created by vesicle motion in the direction of axis of symmetry.

As the membrane opens a pore, the inner content of the vesicle leaks out and the vesicle shrinks. We do not observe any vesicle swelling in our experiments, therefore, we neglect the solvent flow into the vesicle due to osmotic imbalance. Thus, we can relate the rate of vesicle shrinkage to the leak out of inner content through pore opening as

$$A_{\mathrm{sph}}\dot{R} = -q_{\mathrm{out}}A_{\mathrm{p}}, \tag{S10}$$

where q_{out} is efflux of inner content through the pore and $A_p = \pi R^2\sin^2\alpha$ is the area of the circular pore. The pressure difference Δp_1 govern the solvent efflux as

$$q_{\mathrm{out}} = \frac{\pi\Delta p_1 R\sin\alpha}{Q\eta_{\mathrm{s}}}, \tag{S11}$$

where the geometric coefficient $Q = (1+2\cos\alpha)\sin^2\alpha + \frac{1}{2}(\pi-\alpha)(11+\cos\alpha)\sin\alpha + 3(\pi-\alpha)^2(2-\cos\alpha)\cos^2\frac{\alpha}{2}$, generalizes the Sampson relationship (20) for an unbounded fluid passing through a circular hole in an infinite plane wall to the case of fluid passing through the a hole in a bounded hollow sphere (5). Now, substituting Eq. S11 in Eq. S10, gives

$$-\Delta p_1 = \frac{Q\eta_{\mathrm{s}}}{\pi^2 R^3\sin^3\alpha}A_{\mathrm{sph}}\dot{R}. \tag{S12}$$

Now, substituting the Δp_1 from Eq. S12 in Eq. S9, gives

$$\Phi_{\mathrm{a}} = 4\eta_s R\frac{Q(1+\cos\alpha)}{\sin^3\alpha}\dot{R}^2 + \eta_s R\left(C_{\mathrm{a2}}\dot{R}R\dot{\alpha} + \frac{1}{3}C_{\mathrm{a2}}\tan\frac{\alpha}{2}(R\dot{\alpha})^2\right). \quad \text{[S13]}$$

Finally, by writing $C_{\mathrm{a1}} = 4\frac{Q(1+\cos\alpha)}{\sin^3\alpha}$ and $C_{\mathrm{a3}} = \frac{1}{3}C_{\mathrm{a2}}\tan\alpha/2$, we express the viscous losses in the aqueous solution, as a Rayleigh dissipation potential given by

$$\Phi_a = \eta_s R\left(C_{\mathrm{a1}}\dot{R}^2 + C_{\mathrm{a2}}\dot{R}\left(R\dot{\alpha}\right) + C_{\mathrm{a3}}\left(R\dot{\alpha}\right)^2\right), \quad \text{[S14]}$$

In the curling cases, due to material accumulation around the rim, the retracting pore rim in a viscous surrounding solution dissipates energy. The retracting pore rim is usually modeled as a cylinder moving in a viscous fluid (3, 13). Therefore, we write the Rayleigh dissipation potential to account for viscous dissipation in the aqueous solution due to motion of rim as

$$\Phi_{\mathrm{r}} = \eta_{\mathrm{s}} R C_{\mathrm{r}}\left(\dot{R}^2 + (R\dot{\alpha})^2\right). \quad \text{[S15]}$$

where $C_{\mathrm{r}} = \frac{4\pi^2\sin\alpha}{\ln\left(2\pi R\sin\alpha/r_{\mathrm{o}}\right)+1/2}$. While writing Eq. S15, we consider pore circumference $2\pi R\sin\alpha$ and radius r_{o} as the length and radius of the model cylinder.

B.1. Dissipation potential of viscous losses in vesicle membrane. In addition, disruption in lipid bilayer as the pore grows and vesicle shrinks due to leak of inner content creates a flow of lipids within the membrane itself (2, 21, 22). Due to the large mobility of lipids in the lipid bilayer, the membrane is considered as a thin fluid sheet (21, 22). To account for viscous losses due to lipids flow within the spherical section of vesicle membrane, we start by writing the mass conservation of this deforming fluid membrane as in ref (21)

$$\dot{\rho} + \rho(\nabla_{\mathrm{s}}.\vec{u}_{\mathrm{s}} - \dot{R}H) = 0. \quad \text{[S16]}$$

Here, $\nabla_{\mathrm{s}} = \nabla - \vec{n}(\vec{n}\cdot\nabla)$ is the surface gradient operator, where $\vec{n}$ is a normal to the membrane and $\vec{u}_{\mathrm{s}} = (u_\theta, u_\phi)$ is velocity field of the lipids within the membrane plane. u_θ, and u_ϕ are components of the velocity field u_s in polar, and azimuthal directions, respectively. A model for both curling and no-curling vesicle explosion is shown in Fig S1(A-B) with embedded coordinate system. In modeling the dissipation due to lipids flow in membrane, we consider the lipid bilayer as incompressible homogeneous viscous fluid $\dot{\rho} = 0$, *i.e* there is no change in the density of the lipids in membrane plane. Consequently, the conservation of mass for lipid flow in deforming membrane is written as

$$\nabla_{\mathrm{s}}.\vec{u}_{\mathrm{s}} - \dot{R}H = 0. \quad \text{[S17]}$$

Due to axis-symmetric nature of the flow, u_ϕ and partial derivatives in azimuthal direction $\partial/\partial\phi$ would vanish. Therefore,

$$\frac{1}{R\sin\theta}\frac{\partial u_\theta\sin\theta}{\partial\theta} = -2\frac{\dot{R}}{R}, \quad \text{[S18]}$$

where Eq. S17 is simplified substituting $H = -2/R$ for a sphere. As shown in Fig S1(A-B), the vesicle maintains the spherical geometry, therefore radius of the vesicle R shrinks or expand uniformly *i.e.* R and $\dot{R}$ do not depend on the angle θ. Consequently, using the boundary condition $u_\theta\big|_{\theta=\pi} = 0$ with Eq. S18, we obtain

$$u_\theta = 2\dot{R}\frac{1+\cos\theta}{\sin\theta}, \qquad \alpha \le \theta \le \pi. \quad \text{[S19]}$$

We note here that the Eq S19 is valid only for a vesicle with a pore *i.e.* when $\alpha > 0$. In a vesicle without a pore, due to incompressibility of lipid membrane, R and $\dot{R}$ would be function of angle θ, and Eq. S19 would not be applicable.

Next, the Rayleigh dissipation function to account for viscous losses in spherical membrane is written as

$$\Phi_{\mathrm{sm}} = \eta_{\mathrm{m}}\int_{A_{\mathrm{sph}}}\boldsymbol{d}:\boldsymbol{d}\,\mathrm{d}A, \quad \text{[S20]}$$

where $\boldsymbol{d} = (\nabla_{\mathrm{s}}\vec{u}_{\mathrm{s}} + (\nabla_{\mathrm{s}}\vec{u}_{\mathrm{s}})^T)/2 - \dot{R}\nabla\vec{n}$ (21). Finally, integrating Eq. S20, gives

$$\Phi_{\mathrm{sm}} = 4\pi\eta_{\mathrm{m}}((1+\cos\alpha) + 2(\frac{1+\cos\alpha}{\sin\alpha})^2 + 8\ln(\sin\frac{\alpha}{2}))\dot{R}^2. \quad \text{[S21]}$$

Writing $C_{\mathrm{sm}} = 4\pi((1+\cos\alpha) + 2(\frac{1+\cos\alpha}{\sin\alpha})^2 + 8\ln(\sin\frac{\alpha}{2}))$, we express the Rayleigh dissipation potential as

$$\Phi_{\mathrm{sm}} = \eta_{\mathrm{m}} C_{\mathrm{sm}}\dot{R}^2. \quad \text{[S22]}$$

Here, η_{m} is the membrane surface viscosity, and C_{sm} is a geometric coefficient, that only depends on the pore angle α. As a consequence of incompressibility of lipid membrane, in Eq. S22 the viscous losses in membrane occurs only when the vesicle shrinks or expands.

Furthermore, following Callan *et. al.* (13), we write the Rayleigh dissipation potential to account for viscous losses associated with the lipid flow in the rim section as

$$\Phi_{\mathrm{rm}} = \eta_{\mathrm{m}} C_{\mathrm{rm}} \left(R\dot{\alpha}\right)^2 . \quad \text{[S23]}$$

where $C_{\mathrm{rm}} = \frac{1}{2}\frac{A_{\mathrm{rim}}}{(R\sin\alpha)^2}$. Therefore, following Eqs. S9, S15, S22, and S23, the total dissipation potential of vesicles explosion in the curling mode is written as

$$\Phi_{\mathrm{c}} = \Phi_{\mathrm{a}} + \Phi_{\mathrm{r}} + \Phi_{\mathrm{sm}} + \Phi_{\mathrm{rm}}. \quad \text{[S24]}$$

However, in cases of no-explosion, due to the absence of material accumulation around the pore edge, the total dissipation potential in no-curling mode of vesicle explosion is given by

$$\Phi_{\mathrm{nc}} = \Phi_{\mathrm{a}} + \Phi_{\mathrm{sm}}. \quad \text{[S25]}$$

C. Governing equations for pore expansion.

C.1. Curling mode of vesicle explosion. Now, employing the Onsager's variational principle (Eq. S1) with Eqs. S7 and S24,

$$\frac{\partial E_{\mathrm{c}}}{\partial R} = -\frac{\partial \Phi_{\mathrm{c}}}{\partial \dot{R}} \quad \text{[S26a]}$$

$$\frac{1}{R}\frac{\partial E_{\mathrm{c}}}{\partial \alpha} = -\frac{\partial \Phi_{\mathrm{nc}}}{\partial (R\dot{\alpha})} , \quad \text{[S26b]}$$

provides

$$2\pi\gamma\sin\alpha - 4\pi k_{\mathrm{b}} H_{\mathrm{s}}\left(1+\cos\alpha\right) + \frac{2\pi^2 k_{\mathrm{b}}\sin\alpha}{l}\left(\ln\frac{r_{\mathrm{o}}}{r_{\mathrm{i}}} - 2|H_{\mathrm{s}}|(r_{\mathrm{o}}-r_{\mathrm{i}}) + R(\frac{1}{r_{\mathrm{o}}} - 2|H_{\mathrm{s}}|)\frac{\partial r_{\mathrm{o}}}{\partial R}\right) = -\eta_{\mathrm{s}} R\left(2(C_{\mathrm{a1}}+C_{\mathrm{r}})\dot{R} + C_{\mathrm{a2}}(R\dot{\alpha})\right) - 2\eta_{\mathrm{m}} C_{\mathrm{sm}}\dot{R} \quad \text{[S27a]}$$

$$2\pi\gamma\cos\alpha - 4\pi k_{\mathrm{b}}(1/R - H_{\mathrm{s}})\sin\alpha + \frac{2\pi^2 k_{\mathrm{b}}}{l}\left(\cos\alpha\left(\ln\frac{r_{\mathrm{o}}}{r_{\mathrm{i}}} - 2|H_{\mathrm{s}}|(r_{\mathrm{o}}-r_{\mathrm{i}})\right) + \sin\alpha(\frac{1}{r_{\mathrm{o}}} - 2|H_{\mathrm{s}}|)\frac{\partial r_{\mathrm{o}}}{\partial \alpha}\right) = -\eta_{\mathrm{s}} R\left(C_{\mathrm{a2}}\dot{R} + 2(C_{\mathrm{a3}}+C_{\mathrm{r}})(R\dot{\alpha})\right) - 2\eta_{\mathrm{m}} C_{\mathrm{rm}}(R\dot{\alpha}), \quad \text{[S27b]}$$

the coupled governing equations for shrinkage of vesicle radius R and expanion of pore angle α in case of vesicle explosion in curling mode.

C.2. No-curling mode of vesicle explosion. Similarly, employing the Onsager's variational principle (Eq. S1) with Eqs. S8 and S25,

$$\frac{\partial E_{\mathrm{nc}}}{\partial R} = -\frac{\partial \Phi_{\mathrm{nc}}}{\partial \dot{R}} \quad \text{[S28a]}$$

$$\frac{1}{R}\frac{\partial E_{\mathrm{nc}}}{\partial \alpha} = -\frac{\partial \Phi_{\mathrm{nc}}}{\partial (R\dot{\alpha})} , \quad \text{[S28b]}$$

gives

$$2\pi k_{\mathrm{b}} H_{\mathrm{s}}\left(H_{\mathrm{s}}R - 2\right)\left(1+\cos\alpha\right) = -\eta_{\mathrm{s}} R\left(2C_{\mathrm{a1}}\dot{R} + Ca_{\mathrm{a2}}(R\dot{\alpha})\right) - 2\eta_{\mathrm{m}} C_{\mathrm{sm}}\dot{R} \quad \text{[S29a]}$$

$$-\pi k_{\mathrm{b}}(4/R - 4H_{\mathrm{s}} + H_{\mathrm{s}}^2 R)\sin\alpha = -\eta_{\mathrm{s}} R\left(C_{\mathrm{a2}}\dot{R} + 2C_{\mathrm{a3}}(R\dot{\alpha})\right), \quad \text{[S29b]}$$

will provide the governing equation of pore dynamics in case of vesicle explosion in no-curling mode.

C.3. Numerical solution of governing equations. To numerically solve the governing equations, we rewrite the coupled equations in matrix form appropriate for the ODE solver. We choose line tension $\gamma = 6$ pN of the DOPC lipid bilayer, obtained from pore closing experiments of phtosensitized GUVs (23). For pure DOPC lipid bilayers, the bending rigidity $k_b \approx 22k_BT$ (24). However, as the lipid oxidation softens the lipid bilayers (25), in our simulations we choose bending rigidity $k_b = 10k_BT$. For solvent viscosity, $\eta_{\mathrm{s}} = 2\times10^{-3}$ Pa·s. Inclusion of oil residue in membrane during vesicle fabrication has been found to change the surface viscosity of the lipid bilayer by an order of magnitude (26). Therefore, in our numerical solutions, we use a value of surface viscosity $\eta_{\mathrm{m}} = 50$ nPa·s·m (for pure DOPC $\eta_{\mathrm{m}} = 4.11 \pm 2.63$ nPa·s· (24)). Since the PS is highly hydrophilic (logP < -3), it does not adsorb into the membrane. Therefore, we do not expect the membrane properties to change with the concentration of the PS. The only remaining unknown parameter is the spontaneous curvature H_{s}, which we determine by minimizing the cost function $\boldsymbol{D}:\boldsymbol{D}$, where $D_{\mathrm{i},1} = \left((R_{\mathrm{n}} - R_{\mathrm{e}})/R_0\right)_{\mathrm{i}}$, and $D_{\mathrm{i},2} = \left((R_{\mathrm{n}}\alpha_{\mathrm{n}} - R_{\mathrm{e}}\alpha_{\mathrm{e}})/R_0\right)_{\mathrm{i}}$. The subscript i denotes the ith time step, and subscripts n and e represent the corresponding numerical and experimental values, respectively.

2. Criteria for vesicle explosion

A. Total energy landscape of the vesicle. In Fig.6D of the main text, we show how the impact of spontaneous curvature on the total energy of a vesicle

$$E = E_{\rm s} + E_{\rm p} + E_{\rm b}, \tag{S30}$$

consisting of stretching energy, pore edge energy, and bending energy of the vesicle. To compute stretching energy $E_{\rm s} = \sigma^2 A_0/2K$, we use the constitutive relation $\varepsilon = \frac{k_{\rm B}T}{8\pi k_b} \ln(1+\sigma/\sigma_{\rm c}) + \frac{\sigma}{K}$, where $\varepsilon = A/A_0 - 1$ is defined as membrane area strain. Here, A is instantaneous membrane area and A_0 is the base membrane area of unstressed membrane. We choose stretching modulus $K = 100$ mN/m for oxidized DOPC (25) and the reference tension state $\sigma_{\rm c} = 0.001$ mN/m of lipid membrane in short-wavelength limit (27, 28).The spontaneous curvature $H_{\rm s}$ causes the budding of smaller vesicles or tubular structures reducing the base membrane area A_0 while the encapsulated vesicle volume remains same. Therefore, these budding-off events create membrane area strain ε. Once the membrane tension σ reaches a critical value, the membrane ruptures and opens a micron-sized pore (2, 4, 6, 7, 29).

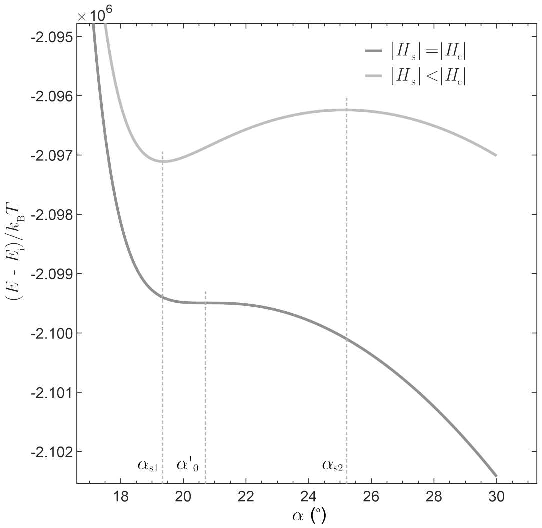

Fig. S2. Total energy profile of a vesicle showing energy barrier between two stationary points $\alpha_{\rm s1}$ and $\alpha_{\rm s2}$ (with respect to α) for cases $|H_{\rm s}| < |H_{\rm c}|$. At $|H_{\rm s}| = |H_{\rm c}|$, the stationary points merge into α_0', and the energy barrier vanishes. $E_{\rm i}$ is the initial energy just before the membrane rupture.

In Fig. S2 and 6D, we show how the spontaneous curvature $H_{\rm s}$ modulates the energy profile $E - E_{\rm i}$ of a vesicle. Here, $E_{\rm i}$ is initial energy of the intact vesicle just before the pore opens. As the pore expands, the membrane area strain is reduced, decreasing the membrane tension. The initial pore expansion is driven by the relaxation of membrane tension (2, 4, 6, 7, 29). In cases, $|H_{\rm s}| < |H_{\rm c}|$ as shown in Fig. S2, the energy profile have two stationary points $\alpha_{\rm s1}$ (given by $\partial E/\partial\alpha = 0,\ \partial^2 E/\partial\alpha^2 > 0$ and $\alpha_{\rm s2}$ (given by $\approx \partial(E_{\rm p} + E_{\rm b})/\partial\alpha = 0,\ \partial^2 E/\partial\alpha^2 < 0$). At $\alpha_{\rm s1}$ the membrane tension is mostly relaxed, therefore, beyond $\alpha_{\rm s1}$ there is competition only between the pore edge energy $E_{\rm p}$ and the bending energy $E_{\rm b}$. After $\alpha_{\rm s2}$, the bending energy dominates and creates a favorable energy gradient for the pore to grow. However, for $|H_{\rm s}| < |H_{\rm c}|$, there exist a energy barrier between the stationary points $\alpha_{\rm s1}$ and $\alpha_{\rm s2}$. Consequently, the pore expands up to α_a and the pore edge energy $E_{\rm p}$ resists any further expansion and reseals the pore. Upon increasing the spontaneous curvature $|H_{\rm s}|$ to $|H_{\rm c}|$, as presented in Fig. S2, the stationary points $\alpha_{\rm s1}$ and $\alpha_{\rm s2}$ merges into α_0' given by $\partial E/\partial\alpha = \partial^2 E/\partial\alpha^2 = 0$ and the energy barrier vanishes. Thus, for $|H_{\rm s}| \geq |H_{\rm c}|$, beyond $\alpha_0' \approx \alpha_0$ estimated as the pore angle at the first image frame, the pore keeps expanding, developing into curling or no-curling mode of vesicle explosion. At α_0, the membrane area strain $\varepsilon \approx 0$. Therefore, the instantaneous area of a vesicle with a pore at this moment is $2\pi R_0^2(1+\cos\alpha_0)$, which is approximated as the area of the unstressed membrane A_0. Here, R_0 is the radius of the vesicle before a pore open. Thus, for a given critical membrane area strain ε_0 ($\approx 4\%$ at which oxidized DOPC vesicles rupture (30)), we can relate it to α_0 as

$$\varepsilon_0 \approx \frac{4\pi R_0^2}{2\pi R_0^2(1+\cos\alpha_0)} - 1 = \frac{2}{(1+\cos\alpha_0)} - 1 \tag{S31}$$

Rearranging Eq. S31, gives the relation $\alpha_0 \approx \cos^{-1}(2/(1+\varepsilon_0) - 1)$ between pore angle α_0 and critical membrane strain ε_0 at membrane rupture as discussed in section "Spontaneous curvature assists vesicle explosion" of main text.

After the rapid initial growth of pore angle until α_0, the further pore expansion or closure is determined by the competition between the pore edge energy $E_{\rm p}$ and the bending energy $E_{\rm b}$. The pore edge energy $E_{\rm p}$ favors the pore closure while the bending energy $E_{\rm b}$ favors the pore expansion. With a favorable gradient of total energy $\partial E/\partial\alpha\big|_{R_0,\alpha_0} < 0$, the bending energy dominates making the pore to grow and develop into curling or no-curling mode of vesicle explosion. In following subsections, we derive the criteria for no-curling and curling modes of vesicle explosion.

B. No-curling. In the no-curling mode, the tubules-like structures are observed to be sprouting around the pore rim. The generation of tubules reduces the line tension γ (17) and hence the effect of pore edge energy E_{p} on pore dynamics is considered to be negligible in the current work. However, before the edge of a transient pore develops into tubules in the no-curling mode of explosion *i.e.* when the pore angle $\alpha \lessapprox \alpha_0$, the pore edge energy E_{p} cannot be neglected. Therefore, the total energy of a vesicle in the no-curling mode of explosion is given by

$$E_{\mathrm{nc}} = 2\pi\gamma R \sin\alpha + \frac{1}{2} k_{\mathrm{b}} \left(H - H_{\mathrm{s}}\right)^2 A_{\mathrm{sph}} \qquad \text{[S32]}$$

From Eq. S32, the condition of favorable energy gradient $\partial E_{\mathrm{nc}}/\partial\alpha\big|_{R_0,\alpha_0} < 0$ gives $2\gamma R_0 \cos\alpha_0 - k_{\mathrm{b}} \left(H - H_{\mathrm{s}}\right)^2 R_0^2 \sin\alpha_0 < 0$. Now, by defining non-dimensional line tension as $\mathit{\Gamma} = \gamma R_0/k_{\mathrm{b}}$ and non-dimensional spontaneous curvature as $\tilde{H} = H_{\mathrm{s}} R_0$ gives $2\mathit{\Gamma} \cos\alpha_0 < \left(2 - \tilde{H}\right)^2 \sin\alpha_0$. Considering $|\tilde{H}| >> 1$ in the current experiments, we obtain

$$|\tilde{H}| \gtrapprox \sqrt{2\mathit{\Gamma} \cot\alpha_0} \qquad \text{[S33]}$$

From Eq. S33, we get the critical non-dimensional spontaneous curvature $|\tilde{H}_{\mathrm{c}}| \approx \sqrt{2\mathit{\Gamma} \cot\alpha_0}$ as in Eq 15 of the main text.

C. Curling. From Eq S7, the condition of favorable energy gradient $\partial E_{\mathrm{c}}/\partial\alpha\big|_{R_0,\alpha_0} < 0$ gives

$$\begin{aligned} &2\pi\gamma R_0 \cos\alpha_0 - 4\pi k_{\mathrm{b}} \left(1 - H_{\mathrm{s}} R_0\right) \sin\alpha_0 + \frac{2\pi^2 k_b R_0 \cos\alpha_0}{l} \left(\ln\left(\frac{r_{\mathrm{o}}}{r_{\mathrm{i}}}\right) - 2|H_{\mathrm{s}}| \left(r_{\mathrm{o}} - r_{\mathrm{i}}\right) \right) \\ &+ \frac{2\pi^2 k_b R_0 \sin\alpha_0}{l} \left(\frac{1}{r_{\mathrm{o}}} - 2|H_{\mathrm{s}}|\right) \frac{\partial r_{\mathrm{o}}}{\partial\alpha}\bigg|_{R_0,\alpha_0} + \frac{1}{2} k_{\mathrm{b}} H_{\mathrm{s}}^2 \left(\frac{\partial A_{\mathrm{sph}}}{\partial\alpha} + \frac{\partial A_{\mathrm{rim}}}{\partial\alpha}\right) < 0 \end{aligned} \qquad \text{[S34]}$$

Now, since $A_0 = A_{\mathrm{sph}} + A_{\mathrm{rim}} = \text{constant}$, and at configuration (R_0, α_0), $r_{\mathrm{o}} = r_{\mathrm{i}} = |H_{\mathrm{s}}|^{-1}$ and $\partial r_{\mathrm{o}}/\partial\alpha\big|_{R_0,\alpha_0} = l R_0 |H_{\mathrm{s}}|/2\pi$, Eq. S34 can be simplified as

$$2\pi\gamma R_0 \cos\alpha_0 - 4\pi k_{\mathrm{b}} \left(1 - H_{\mathrm{s}} R_0\right) \sin\alpha_0 - \pi k_b |H_{\mathrm{s}}|^2 R_0^2 \sin\alpha_0 < 0 \qquad \text{[S35]}$$

Substituting for non-dimensional parameters $\mathit{\Gamma}$ and $\tilde{H}$ in Eq. S35, we obtain $2\mathit{\Gamma} \cos\alpha_0 - \left(4\left(1 - \tilde{H}\right) + \tilde{H}^2\right) \sin\alpha_0 < 0$. Considering large spontaneous curvature such as $\tilde{H}^2 >> |\tilde{H}| >> 1$, Eq. S35 furnishes the relation

$$|\tilde{H}| \gtrapprox \sqrt{2\mathit{\Gamma} \cot\alpha_0}, \qquad \text{[S36]}$$

which is the same as provided by Eq. S33. Therefore, the relation in Eq. 15 (of main text) governs the delineation of vesicle explosion in both modes no-curling as well as curling, and transient pore formation.

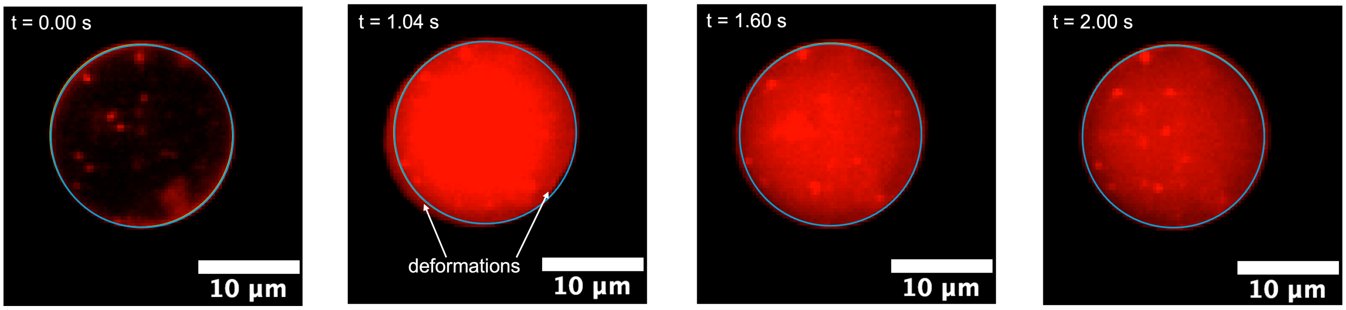

Fig. S3. Shape fluctuation and deformation of a vesicle in the beginning of irradiation. Initially spherical vesicle deforms upon irradiation and returns to the original spherical shape in about a second.

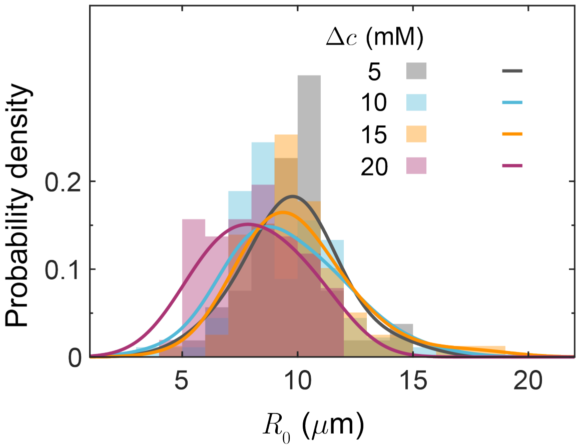

Fig. S4. Size distribution of vesicles irradiated at different concentration differences Δc across membrane. The solid lines shows the probability density function (PDF) plotted using kernel smoothing of the corresponding histogram. Histograms and PDFs for different Δc across membrane shows a similar size distribution of vesicles irradiated to study the impact of oxidation on vesicle stability and integrity.

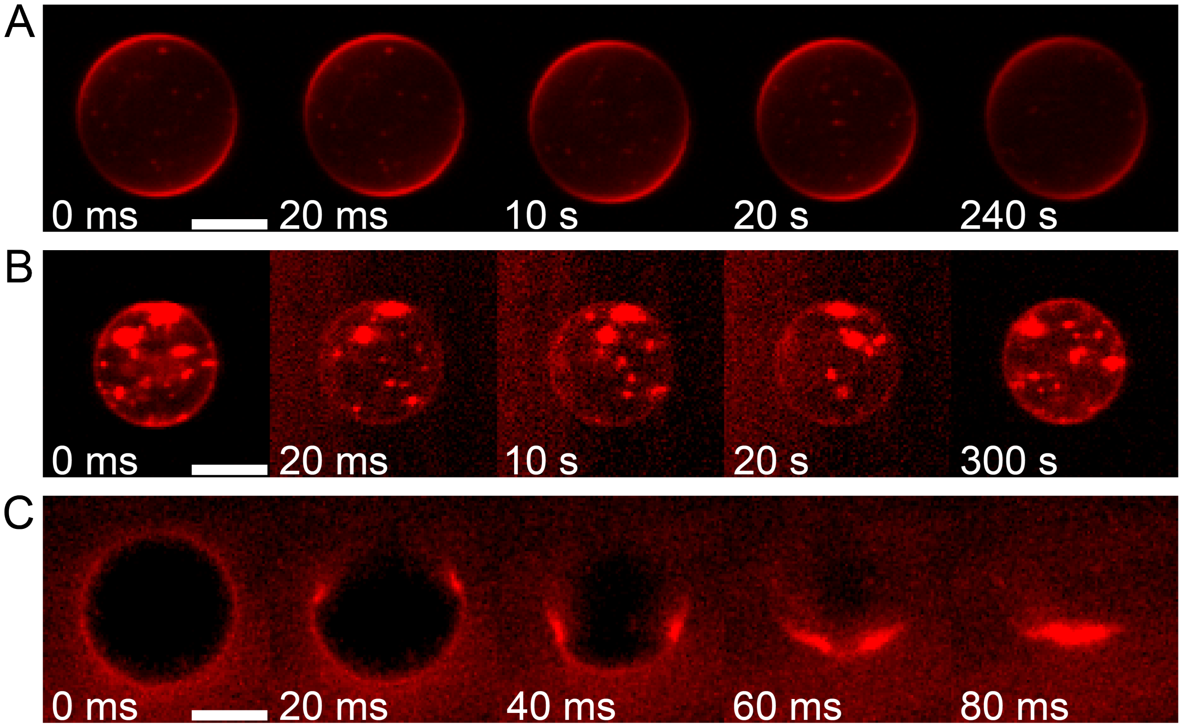

Fig. S5. Vesicle irradiation for symmetric and asymmetric distribution of photosensitizer (PS). *(A)* PS absent ($\Delta c = 0$ mM, $c_{\rm in} = c_{\rm out} = 0$ mM). *(B)* PS symmetrically present inside and outside of vesicle ($\Delta c = 0$ mM, $c_{\rm in} = c_{\rm out} = 20$ mM). *(C)* PS asymmetrically present only outside of vesicle ($\Delta c = -20$ mM, $c_{\rm in} = 0$ mM, and $c_{\rm out} = 20$ mM). In *(A)* and *(B)*, the irradiation starts at $t = 0$ ms, while in (c), $t = 0$ ms just before the moment the membrane ruptures. Later, the HPTS signal subsides, while the fluorescent signal could be a mixed signal from the lipid label and HPTS. Additionally, we note that HPTS is not expected to be absorbed inside the membrane layer due to the polar nature and high solubility of HPTS in aqueous solutions (31, 32).

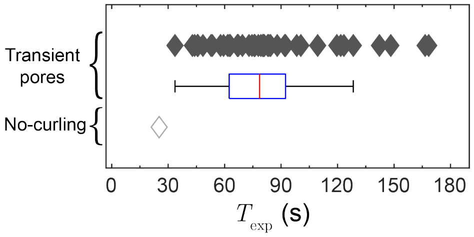

Fig. S6. Vesicle exposure time $T_{\rm exp}$ at $\Delta c = 5$ mM for continuous exposure of 180 s.

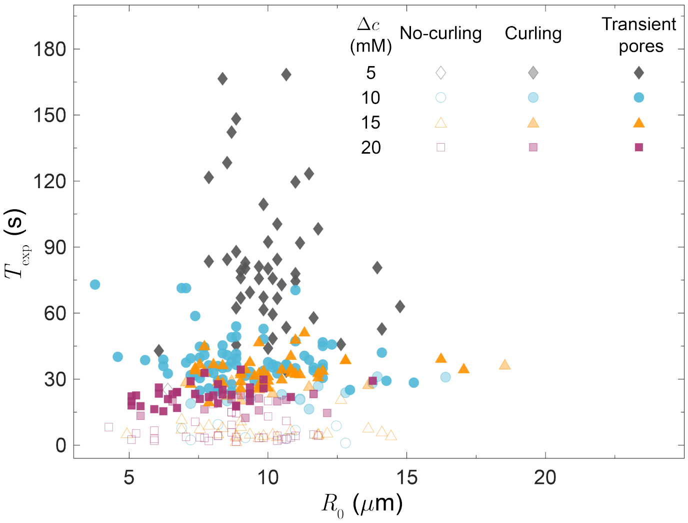

Fig. S7. Vesicle exposure time $T_{\rm exp}$ for different sizes of vesicle. Exposure time $T_{\rm exp}$ of vesicle rupture shows only weak correlation with the vesicle size for experiments performed at different concentration differences Δc. The Pearson correlation coefficients are -0.09 (p = 0.54), -0.21 (p = 0.04), 0.12 (p = 0.29), and -0.03 (p = 0.79) for $\Delta c = 5$, 10, 15, and 20 mM, respectively.

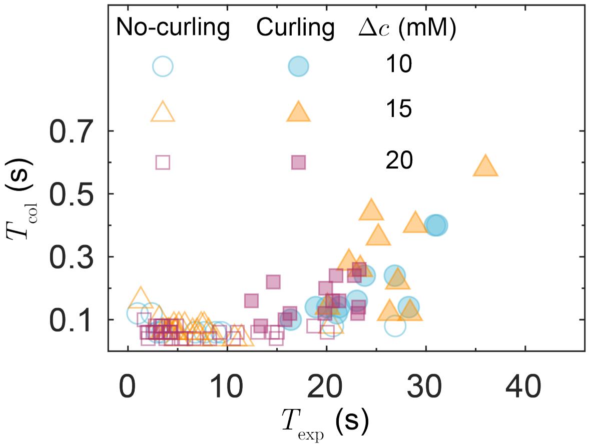

Fig. S8. Vesicle collapse time T_{col} (time duration from moment of membrane rupture until vesicle flattens) with the exposure time T_{exp} (time duration from the start of the radiation until membrane ruptures) for no-curling and curling mode of vesicle explosion at different concentration differences $\Delta c =$ 10, 15, and 20 mM. The vesicle in no-curling mode collapses with in 100 ms ($T_{\mathrm{col}} \lesssim 100$ ms) while the vesicle collapse time in curling mode is generally longer than the 100 ms. Similarly, T_{exp} for vesicle membrane rupture in no-curling mode explosion is shorter than the T_{exp} for membrane rupture in curling mode explosion. In curling mode of vesicle explosion for a increasing T_{exp} of membrane rupture, the T_{col} also increases at all concentration differences Δc.

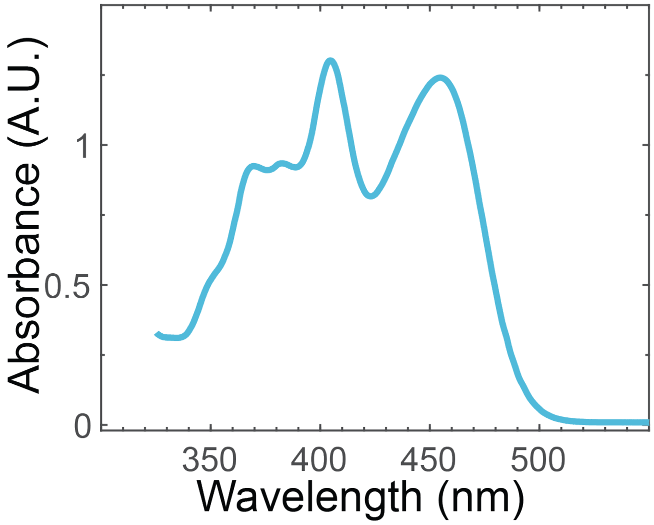

Fig. S9. Electronic absorbance spectrum of a 100 μM HPTS in 500 mM sucrose solution. HPTS was excited with a diode laser at 405 nm.

Table S1. Number of vesicles affected under irradiation used in plotting Fig. 3A of main text.

$\Delta c = c_{\mathrm{in}} - c_{\mathrm{out}}$	Explosion	Transient Pore Formation	Intact	Total no. of vesicles
5 mM	1	24	28	53
10 mM	16	74	0	90
15 mM	35	44	0	79
20 mM	36	15	0	51

Supporting Movies

In the following SI movies, at the beginning of illumination, the strong fluorescence signal from PS (HPTS) saturates the channel for lipid labels in the confocal microscopy, and therefore the entire interior of the vesicle is bright. Later, the HPTS signal subsides, while the fluorescent signal could be a mixed signal from the lipid label and HPTS. Additionally, we note that HPTS is not expected to be absorbed inside the membrane layer due to the polar nature and high solubility of HPTS in aqueous solutions (31, 32).

Movie S1. Confocal movie showing the initial shape fluctuation upon irradiation of a vesicle encapsulating a PS concentration of 10 mM. After about a second, the the vesicle returns to the original spherical shape.

Movie S2. Real-time movie shows the budding off of a tubule from the mother vesicle encapsulating a PS concentration of 15 mM. Subsequent to the budding event, the vesicle opens a transient pore.

Movie S3. Real-time movie shows the budding off of a tubule from the mother vesicle encapsulating a PS concentration of 10 mM. After the budding of tubule, the vesicle opens a pore and explodes in the curling mode.

References

1. M Arroyo, N Walani, A Torres-Sánchez, D Kaurin, Onsager's variational principle in soft matter: introduction and application to the dynamics of adsorption of proteins onto fluid membranes. *The Role Mech. Study Lipid Bilayers* pp. 287–332 (2018).
2. O Sandre, L Moreaux, F Brochard-Wyart, Dynamics of transient pores in stretched vesicles. *Proc. Natl. Acad. Sci.* **96**, 10591–10596 (1999).
3. E Mabrouk, D Cuvelier, F Brochard-Wyart, P Nassoy, MH Li, Bursting of sensitive polymersomes induced by curling. *Proc. Natl. Acad. Sci.* **106**, 7294–7298 (2009).
4. M Chabanon, JC Ho, B Liedberg, AN Parikh, P Rangamani, Pulsatile lipid vesicles under osmotic stress. *Biophys. J.* **112**, 1682–1691 (2017).
5. RJ Ryham, On the viscous flows of leak-out and spherical cap natation. *J. Fluid Mech.* **836**, 502–531 (2018).
6. VK Malik, S Shin, J Feng, Light-triggered explosion of lipid vesicles. *Soft Matter* **16**, 8904–8911 (2020).
7. VK Malik, OS Pak, J Feng, Pore dynamics of lipid vesicles under light-induced osmotic stress. *Phys. Rev. Appl.* **17**, 024032 (2022).
8. J Heuvingh, S Bonneau, Asymmetric oxidation of giant vesicles triggers curvature-associated shape transition and permeabilization. *Biophys. J.* **97**, 2904–2912 (2009).
9. R Dasgupta, MS Miettinen, N Fricke, R Lipowsky, R Dimova, The glycolipid gm1 reshapes asymmetric biomembranes and giant vesicles by curvature generation. *Proc. Natl. Acad. Sci.* **115**, 5756–5761 (2018).
10. Y Dreher, K Jahnke, M Schroter, K Gopfrich, Light-triggered cargo loading and division of dna-containing giant unilamellar lipid vesicles. *Nano Lett.* **21**, 5952–5957 (2021).
11. D Kabaso, R Shlomovitz, T Auth, VL Lew, NS Gov, Curling and local shape changes of red blood cell membranes driven by cytoskeletal reorganization. *Biophys. J.* **99**, 808–816 (2010).
12. M Abkarian, G Massiera, L Berry, M Roques, C Braun-Breton, A novel mechanism for egress of malarial parasites from red blood cells. *Blood* **117**, 4118–4124 (2011).
13. A Callan-Jones, OEA Arriagada, G Massiera, V Lorman, M Abkarian, Red blood cell membrane dynamics during malaria parasite egress. *Biophys. J.* **103**, 2475–2483 (2012).
14. Z Tu, Z Ou-Yang, Lipid membranes with free edges. *Phys. Rev. E* **68**, 061915 (2003).
15. N Kučerka, S Tristram-Nagle, JF Nagle, Structure of fully hydrated fluid phase lipid bilayers with monounsaturated chains. *J. Membr. Biol.* **208**, 193–202 (2006).
16. RB Lira, FS Leomil, RJ Melo, KA Riske, R Dimova, To close or to collapse: the role of charges on membrane stability upon pore formation. *Adv. Sci.* **8**, 2004068 (2021).
17. M Aleksanyan, RB Lira, J Steinkühler, R Dimova, Gm1 asymmetry in the membrane stabilizes pores. *Biophys. J.* **121**, 3295–3302 (2022).
18. R Ryham, I Berezovik, FS Cohen, Aqueous viscosity is the primary source of friction in lipidic pore dynamics. *Biophys. J.* **101**, 2929–2938 (2011).
19. CA Aubin, RJ Ryham, Stokes flow for a shrinking pore. *J. Fluid Mech.* **788**, 228–245 (2016).
20. J Happel, H Brenner, *Low Reynolds number hydrodynamics: with special applications to particulate media.* (Springer Science & Business Media) Vol. 1, (1983).
21. M Arroyo, A DeSimone, Relaxation dynamics of fluid membranes. *Phys. Rev. E* **79**, 031915 (2009).
22. A Torres-Sánchez, D Millán, M Arroyo, Modelling fluid deformable surfaces with an emphasis on biological interfaces. *J. Fluid Mech.* **872**, 218–271 (2019).
23. S Sankhagowit, et al., The dynamics of giant unilamellar vesicle oxidation probed by morphological transitions. *Biochimica et Biophys. Acta (BBA)-Biomembranes* **1838**, 2615–2624 (2014).

24. HA Faizi, R Dimova, PM Vlahovska, A vesicle microrheometer for high-throughput viscosity measurements of lipid and polymer membranes. *Biophys. J.* **121**, 910–918 (2022).
25. G Weber, et al., Lipid oxidation induces structural changes in biomimetic membranes. *Soft Matter* **10**, 4241–4247 (2014).
26. HA Faizi, A Tsui, R Dimova, PM Vlahovska, Bending rigidity, capacitance, and shear viscosity of giant vesicle membranes prepared by spontaneous swelling, electroformation, gel-assisted, and phase transfer methods: a comparative study. *Langmuir* **38**, 10548–10557 (2022).
27. W Rawicz, KC Olbrich, T McIntosh, D Needham, E Evans, Effect of chain length and unsaturation on elasticity of lipid bilayers. *Biophys. J.* **79**, 328–339 (2000).
28. E Evans, W Rawicz, B Smith, Concluding remarks back to the future: mechanics and thermodynamics of lipid biomembranes. *Faraday Discuss.* **161**, 591–611 (2013).
29. E Karatekin, et al., Cascades of transient pores in giant vesicles: line tension and transport. *Biophys. J.* **84**, 1734–1749 (2003).
30. A Bour, et al., Lipid unsaturation properties govern the sensitivity of membranes to photoinduced oxidative stress. *Biophys. J.* **116**, 910–920 (2019).
31. A Aspée, et al., Reaction kinetics of phenolic antioxidants toward photoinduced pyranine free radicals in biological models. *The J. Phys. Chem. B* **121**, 6331–6340 (2017).
32. Q Liu, M Taniguchi, S Goel, JS Lindsey, Rapid screening of dyes for self-aggregation, adsorption and metabolic integrity–quantitative metrics as a prelude to biological studies. *Dye. Pigment.* **223**, 111914 (2024).
